# Microbial contamination of medicines, medical devices, cosmetics, child and personal care products: a comprehensive review of secondary contamination risks in home-use settings

**DOI:** 10.3389/fmicb.2026.1836448

**Published:** 2026-05-21

**Authors:** Katia Iskandar, Loïc Marchin, Christine Roques

**Affiliations:** 1 Department of Biomedical Sciences, School of Pharmacy, Lebanese International University, Beirut, Lebanon; 2 Department of Health and Social Work, School of Public Health, Lebanese University, Fanar, Lebanon; 3 Center for Collaborative Research Initiatives in Public Health, Higher Institute of Public Health, Saint Joseph University of Beirut, Beirut, Lebanon; 4 Pylote SAS, Dremil-Lafage, France; 5 Laboratoire de Génie Chimique, CNRS, INPT, UPS, Faculté de Pharmacie, Université de Toulouse, Toulouse, France; 6 ACM Pharma FONDEREPHAR, Toulouse, France

**Keywords:** biofilm, home-use products, microbial contamination, patient safety, post-marketing surveillance, secondary contamination

## Abstract

While various compliance with Good Manufacturing Practice and regulations ensures the safety and quality of manufactured products across the supply chain, the transition from controlled environment to home use represents a lurking hazard for microbial contamination, particularly among vulnerable populations. This comprehensive review examines the nature, extent, and clinical significance of microbial contamination in home-use medicines, medical devices, cosmetics, and personal care products, identifying common patterns and prevention strategies across product categories. Literature searches of PubMed, Web of Science, and Google Scholar identified studies examining secondary contamination of consumer-use products applied to skin and mucous membranes. Five categories were analyzed: medicines (eye drops, nasal irrigation devices), medical devices (nebulizers, breast pumps), infant care equipment (feeding bottles, pacifiers), cosmetics (mascara, lipsticks, eyeliners), and personal care products (contact lens cases, toothbrushes). Results showed that contamination rates ranged from 2 to 100% across products despite quality control and stringent regulation oversights. The predominant microbial contaminants were *Pseudomonas aeruginosa*, *Enterobacter* spp., *Staphylococcus aureus*, fungi, and molds. Biofilm formation was ubiquitous across nebulizers, contact lens cases, and feeding equipment, despite reported compliance with manufacturer instructions. Vulnerable individuals, including immunocompromised individuals, neonates, and elderly persons, are exposed to serious risk, including keratitis, respiratory exacerbations, and neonatal sepsis. These findings indicate that post-marketing contamination represents a critical regulatory gap between manufacturing controls and home-use. Addressing this hazard requires integrated strategies, including innovative product designs, antimicrobial surface technologies, standardized evidence-based hygiene protocols, post-market surveillance systems, and targeted public health interventions, accounting for socioeconomic barriers and health literacy disparities.

## 1 Introduction

Microbial contamination is a significant hazard to various industries, including the production of medicines, medical devices, cosmetics, and personal care products (Tropea, 2022; da Silva et al., 2025; Tyski et al., 2025; Gupta et al., 2024; Roy et al., 2023; Osuoha et al., 2023). It is a well-documented risk throughout the supply chain (Singh et al., 2016; Jaberidoost et al., 2013; Kaple, 2020; Ardi et al., 2024). To mitigate this problem, industry compliance with Good Manufacturing Practices (GMP) guidelines remains central (Khanna, 2018; He et al., 2015; European Medicines Agency, 2022; Food and Drug Administration, 2025; World Health organization, n.d.). GMPs are production standards designed to secure the quality and safety of medicines, medical devices, cosmetic products, food, and dietary supplements (Food and Drug Administration, 2025). Preventive measures include the use of preservatives, microbiological testing, training end-users, preventing cross-contamination, and adequate packaging systems (da Silva et al., 2025; Tyski et al., 2025; Roy et al., 2023). While regulatory oversight has successfully minimized microbial contamination of pharmaceuticals, medical devices, and cosmetic products across the supply chain, this risk remains an overlooked threat to consumer safety during use (Roy et al., 2023; Cunningham-Oakes et al., 2019).

**Figure 1:**
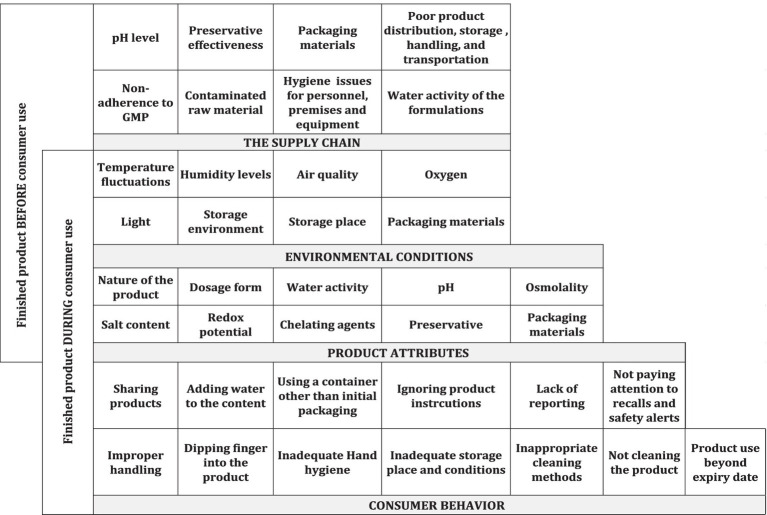
Risks of microbial contamination across the supply chain and during home use.

The transition from controlled manufacturing environments to diverse home settings introduces hazards with varying hygiene practices, storage conditions, environmental microbial loads, and user knowledge levels (Tyski et al., 2025; Roy et al., 2023; Akhand et al., 2023; Michalek et al., 2019; Figure 1).

Home-use products, including medicines, medical devices, cosmetics, and personal care products, can become contaminated through inadequate handling and cleaning techniques by users (Tyski et al., 2025; Roy et al., 2023; Akhand et al., 2023; Michalek et al., 2019). This post-marketing contamination represents a potentially significant yet understudied threat to patient safety.

Product contamination typically involves susceptible and resistant bacteria and fungi, posing serious health risks, particularly to vulnerable individuals, including children, the elderly, and immunocompromised patients (Tropea, 2022; da Silva et al., 2025; Tyski et al., 2025; Gupta et al., 2024; Roy et al., 2023). Beyond the well-documented direct health impacts, the consequences include substantial economic costs due to recalls, medical costs, lawsuits, regulatory penalties and erosion of public trust (Jimenez, 2019; Miglani et al., 2022; Gupta et al., 2024).

Despite its global significance in routine microbiology, the risks associated with contaminated medicines, medical devices, cosmetics, and personal care products are poorly characterized, with limited literature available on the subject (Tropea, 2022; da Silva et al., 2025; Tyski et al., 2025; Cunningham-Oakes et al., 2019; Akhand et al., 2023; Michalek et al., 2019; Jimenez, 2019). This study aims to elucidate the hazard of microbial contamination in home settings by investigating the nature and extent of microbial contamination of selected home-use products and examining the potential risks and their clinical significance on consumers’ safety.

## 2 Search strategy

### 2.1 Search methodology

A comprehensive narrative literature review was conducted to examine microbial contamination of home-use medicines, medical devices, cosmetics and personal care products intended for application to the skin and mucous membranes. The search strategy encompassed three electronic databases: PubMed, Web of Science, and Google Scholar. The search was structured around three primary concepts based on keywords combined using Boolean operators. The first concept addressed microbial contamination using the keywords: microbial contamination, microbiological contamination, bacterial contamination, fungal contamination, mold and yeast contamination, microbial load, and microbial proliferation. The second concept focused on home-use settings, incorporating key terms including home settings, home environment, household, and household environment. The third concept encompassed product types, including: pharmaceuticals, sterile and non-sterile drugs, medicines, medications, medical devices, cosmetics, personal care products, and infant care products intended for application to or contact with the skin and mucous membranes.

Applied filters included human subjects and publications written in English, with no restrictions on publication dates. Both peer-reviewed journal articles and gray literature sources, including government reports, industry publications, and dissertations, were considered for potential inclusion. The search specifically targeted microbial contamination occurring during consumer use and storage. Studies were included if they examined products collected from actual consumer environments after use. Exclusion criteria were products for professional or clinical use only, single-use sterile devices, products with no skin or mucous membrane contact, food products, and studies examining only chemical stability without microbiological assessment.

The Boolean search string used in PubMed was: (microbial contamination OR microbiological contamination OR bacterial contamination OR fungal contamination OR mold and yeast contamination OR microbial load OR microbial proliferation) AND (home settings OR home environment OR household OR household environment) AND (pharmaceuticals OR sterile drugs OR non-sterile drugs OR medicines OR medications OR medical devices OR cosmetics OR personal care products OR infant care products).

### 2.2 Rationale of products selection

The selection of product categories was based on several converging factors for scientific rigor and public health relevance. First, products required documented evidence of microbial contamination in peer-reviewed literature, ensuring the review synthesized established rather than theoretical risks. Second, inclusion prioritized products with direct mucous membrane contact or use on compromised skin sites. Third, selection focused on products frequently used by susceptible groups, such as immunocompromised individuals, neonates, the elderly, and patients with chronic diseases, for whom microbial exposure increases the risk of infection. Fourth, the product spectrum encompasses diverse regulatory classes (medicines, medical devices, cosmetics, and personal care items) allowing comparative analysis of contamination patterns across industry standards and preservation categories.

Based on these criteria, five major product categories examined: (1) sterile medicines (i.e., eye drops, nasal irrigation devices); (2) medical devices for respiratory and neonatal care (i.e., nebulizers, breast pumps); (3) infant care equipment (i.e., feeding bottles, pacifiers); (4) cosmetics intended for application to the eyes and mouth (i.e., mascara, eyeliners, lipsticks, foundations) and (5) personal hygiene items (i.e., contact lens cases, toothbrushes).

Microbial contamination may occur during the manufacturing process (primary contamination), along the supply chain, and during consumer use (secondary contamination; Bhattacharyya and Kepnes, 2002; Welch et al., 2009). While manufacturing controls and regulatory frameworks establish stringent quality standards at the point of production, the following section examines the secondary contamination that occurs when products transition from controlled manufacturing environments to diverse home settings.

## 3 Microbial contamination of home-use products

This section presents secondary contamination patterns, isolated microorganisms, associated risk factors, and clinical significance for each product category.

### 3.1 Medicines

#### 3.1.1 Eye drops

Eye drops should ideally remain sterile throughout the supply chain and during handling by the patient or consumer (Roquefeuil et al., 2024; Iskandar et al., 2022). The microbial contamination rate of preserved and preservative-free in-use eye drops is estimated to be between 2.3 and 73% (Iskandar et al., 2022). Microbial contamination of home-use eye drops is documented in multiple studies (Schein et al., 1992; Donzis, 1997; Porges et al., 2004; Kim et al., 2008; López-García and García-Lozano, 2012; Teuchner et al., 2015; Nisar et al., 2017; Kyei et al., 2019; Chantra et al., 2022). A 30-year literature review highlighted that microbial contamination of the dropper tip and cap of in-use eye drops is from 7.7 to 100% of the total contaminated samples (Iskandar et al., 2022). In this review, the contamination rates of home-used eye drops ranged from 2 to 100%, with most studies finding rates between 13 and 29% (Iskandar et al., 2022). Based on the data in Table 1, preservative-free eye drops showed contamination rates ranging from 29 to 100%, while preserved formulations ranged from 2 to 94%. When specified, the dropper tip and the medication content were the most commonly contaminated parts of the eye drop containers. Contaminants included bacteria, yeasts, and molds that were isolated mainly from the dropper tip, followed by the drops and residual content, even in eye drops containing preservatives (Schein et al., 1992; Donzis, 1997; Porges et al., 2004; Kim et al., 2008; López-García and García-Lozano, 2012; Teuchner et al., 2015; Nisar et al., 2017; Kyei et al., 2019; Chantra et al., 2022; Iskandar et al., 2022). Although documented bacterial contamination is part of the commensal flora and the environment, potential pathogenic bacteria, such as *Pseudomonas aeruginosa*, *Staphylococcus aureus*, *Corynebacterium* spp., *Stenotrophomonas maltophilia*, and *Serratia marcescens*, were also isolated from tested samples (Schein et al., 1992; Donzis, 1997; Porges et al., 2004; Kim et al., 2008; López-García and García-Lozano, 2012; Teuchner et al., 2015; Nisar et al., 2017; Kyei et al., 2019; Chantra et al., 2022; Iskandar et al., 2022). Factors, including improper eye drop administration techniques, extended duration of use, frequency of use, and physical difficulties such as loss of dexterity during instillation related to older age or incapacities, may increase the risk of microbial contamination (Iskandar et al., 2022). Close attention should be paid to the possible contamination mechanisms of the dropper tip and cap, as they may act as potential reservoirs and contribute to re-infection and cross-contamination (Iskandar et al., 2022; Table 1).

**Table 1:** Documented microbial contamination of home-use eye drops.

Medication used	Rate of MC	Duration of use	Site of MC	Detected microorganisms			Ref.
				GPB	GNB	Fungi	
Aerosol saline*	100%		Dropper tip, content		*P*. *aeruginosa*		Rothstein et al. (2019)
Steroids, Antibiotics, Lubricants, Anti-glaucoma eye drops	29%	2 months	Cap, content	CoNS, *Propionibacterium* spp., Diphteroids spp.	Not specified		Schein et al. (1992)
Anti-glaucoma eye drops	13%		Dropper tip, content	*S*. *epidermidis*, *S*. *viridans*	*P*.*aeruginosa*, *Klebsiella* spp.		Porges et al. (2004)
Artificial tears*	2%	10 h	Content	CoNS, *Propionibacterium* spp., *Diphteroids* spp.	*A*. *baumannii*		Kim et al. (2008)
Autologous serum*	29%	7 days	Content	*CoNS*, *S*. *aureus*, *S*. *epidermidis*, *S*. *pneumoniae*, *S*. *pyogenes*	*Pantoea* spp., *P*. *fluorescens*, *S*. *maltophilia*, *Arthrobacter* spp.	*C*. *parapsilosis*	López-García and García-Lozano (2012)
Anit-glaucoma	24.40%	4 weeks	Dropper tip, content	*Staphylococcus* spp. *non-aureus*, *M*. *luteus*, *Corynebacterium* spp., *Bacillus* spp., *R*. *dentocariosa*	*P*. *aeruginosa*, *Pseudomonas* spp., *S*. *maltophylia*, *S*. *marcescens*, *Neisseria* spp.		Teuchner et al. (2015)
Antibiotics, Non-steroidal anti-inflammatory drugs, lubricants, antiallergy, steroids eye drops	23.00%		Content	*S*. *aureus*, *Bacillus* spp.	*P*. *aeruginosa*, *Klebsiella* spp., *K*. *pneumoniae*, *Enterobacter* spp., *P*. *mirabilis*		Nisar et al. (2017)
Anti-infective, anti-glaucoma, anti-allergic, anti-inflammatory eye drops	94%	2 weeks	Content	*Staphylococcus* spp., *S*. *aureus*, *Bacillus* spp.	*Pseudomonas* spp., *Salmonella* spp., *Enterobacter* spp., *E*. *coli*, *Serratia* spp., *S*. *marcescens*, *Shigella* spp., *Proteus* spp.	*Aspergillus* spp., *Penicillium* spp., *Cladosporium* spp., *and Alternaria* spp.	Kyei et al. (2019)
Antibiotics*, Anitfungals*		> 2 days	Dropper tip, content	*S*. *lugdunensis*, *S*. *epidermidis*, *M*. *luteus*, *B*. *cereus*, *K*. *rhizophila*	*Acinetobacte*r spp., *P*. *aeruginosa*, *Arthrobacter*, *B*. *casei*, *K*. *rhizophila*	*Trichosporon asahii*, Yeats not *Candida*, *Candida* spp., Mold unspecified	Chantra et al. (2022)

*Preservative-free. All eye drops were multi-dose products MC: microbial contamination.

Clinical significance:

Microbial contamination of eye drops is a leading cause of potentially avoidable ocular infection (Templeton III et al., 1982; Wasson et al., 1988; Mayo et al., 1987; Penland and Wilhelmus, 1996; Mah-Sadorra et al., 2005; Tsegaw et al., 2017), including keratitis (Templeton III et al., 1982; Wasson et al., 1988), and corneal ulcer (Mayo et al., 1987; Mah-Sadorra et al., 2005). Contamination of eye drops content, tip, and cap with the above-listed pathogens is particularly harmful to susceptible individuals such as immunocompromised people (including neonates), those who underwent an ophthalmic surgical intervention, extensive contact-lens wearers, individuals with preexisting ocular diseases and lid deformities, and those using topical ophthalmic or systemic steroids (Iskandar et al., 2022).

#### 3.1.2 Inhalers and nasal irrigation products

Metered-dose inhalers and nasal irrigation bottles are the mainstays for drug delivery and sinus irrigation intended to treat a plethora of disease conditions, including chronic rhinosinusitis (CRS), allergic rhinitis, and vulnerable individuals with various underlying pathological conditions. Microbial contamination of home-use devices ranged between 18 and 97% (Bhattacharyya and Kepnes, 2002; Welch et al., 2009; Keen et al., 2010; Lee et al., 2010; Lewenza et al., 2010; Foreman and Wormald, 2011; Foreman et al., 2011; Tan et al., 2014; Zahedi et al., 2019; Nguyen et al., 2019), while concurrent evidence of microbial device contamination and nasal cavity varied between 20 and 72% (Lee et al., 2010; Foreman and Wormald, 2011; Foreman et al., 2011; Tan et al., 2014). A limited number of studies examined the cross-contamination between the nasal cavity and medication devices (Lee et al., 2010; Foreman and Wormald, 2011; Foreman et al., 2011; Tan et al., 2014; Zahedi et al., 2019). Tan et al. (2014) compared device contamination to nasal cavity colonization, finding higher rates of contamination in the devices than in the nasal cavities. Studies indicated that microbial contamination of nasal medication delivery devices was predominantly detected at the nozzle, bottle tip, or valve level (Bhattacharyya and Kepnes, 2002; Tan et al., 2014; Zahedi et al., 2019).

Commonly isolated pathogenic and opportunistic contaminants from used bottles included *Corynebacterium* spp. (Lewenza et al., 2010; Foreman and Wormald, 2011; Foreman et al., 2011), *Bacillus anthracis* (Lewenza et al., 2010), *Rothia dentocariosa* (Lewenza et al., 2010), *Capnocytophaga sputigena* (Lewenza et al., 2010), *and Staphylococcus* spp. (Lee et al., 2010; Lewenza et al., 2010; Tan et al., 2014). Isolated Gram- Negative Bacteria (GNB) encompassed *Pseudomonas* spp. (Lewenza et al., 2010; Foreman et al., 2011; Zahedi et al., 2019), including *P*. *aeruginosa* (Lee et al., 2010; Lewenza et al., 2010; Keen et al., 2010; Foreman and Wormald, 2011; Foreman et al., 2011), *Acinetobacter* spp. (Lewenza et al., 2010; Foreman and Wormald, 2011; Foreman et al., 2011; Zahedi et al., 2019), *Stenotrophomonas* spp. (Lee et al., 2010; Lewenza et al., 2010), and *Sphingomonas* spp. (Foreman and Wormald, 2011; Foreman et al., 2011; Lewenza et al., 2010). Nguyen reported the predominance of coagulase-negative *staphylococci* (CoNS), acid-fast bacilli, *S*. *aureus*, and *Pseudomonas* spp. in tested devices (Nguyen et al., 2019). In this study, the participants had allergic rhinitis, while in other studies, they had CRS or underwent Endoscopic Sinus Surgery (ESS; Nguyen et al., 2019).

The factors contributing to microbial contamination of nasal drug delivery devices include the duration of use, degree of compliance with hygiene instructions, frequency of device replacement, and design (Welch et al., 2009; Lewenza et al., 2010; Keen et al., 2010; Foreman et al., 2011; Tan et al., 2014; Nguyen et al., 2019; Zahedi et al., 2019; Psaltis et al., 2012). The correlation between the duration of use and the degree of contamination is controversial (Foreman et al., 2011; Lewenza et al., 2010; Nguyen et al., 2019; Tan et al., 2014; Keen et al., 2010; Welch et al., 2009; Zahedi et al., 2019; Psaltis et al., 2012) and may be justified by the use of antibiotics during the study period (Nguyen et al., 2019). Although regular cleaning and periodic replacement of all devices (3 months post-use) are recommended (Psaltis et al., 2012), compliance with instructions did not lower the risk of microbial contamination (Lee et al., 2010; Keen et al., 2010; Psaltis et al., 2012). Foreman et al. (2011) evaluated the effect of the device design and showed that squeeze bottles with valves were most contaminated at the valve level (Foreman and Wormald, 2011; Foreman et al., 2011). A recent review highlighted the possibility of geographical differences in the ubiquitous occurrence of bacteria (Psaltis et al., 2012). Hydrophilic *Pseudomonas* spp. are predominant in North American studies, and *S*. *aureus* is most prevalent in Australia (Psaltis et al., 2012; Table 2).

**Table 2:** Documented microbial contamination of home-use medicines.

Disease	Medicine	Bottle type	Rate of MC	Nasal cavity swab	Cultured site	Detected microorganisms				Ref.
						GPB	GNB	Fungi	Flora	
Chronic rhinosinusitis	Nasal steroid inhaler		45%	No	Tip	CoNS*, *Bacillus* spp.			Oral flora	Bhattacharyya and Kepnes (2002)
Post endoscopic sinus surgery	Saline nasal irrigation	Squeeze bottle	29%	No	Bottle reservoir, bottle cap, and bottle tube	*Corynebacterium* spp.	*P*. aeruginosa*, *A*. baumanii*, *A*. *calcoaceticus*, *K*. pneumoniae*, *Enterobacter* spp., *E*. *coli*, *P*. *penneri*, *P*. *stuzeri*, *P*. *actinobacillus*, *A*. *hydrophila*,		Oropharyngeal flora, Skin flora	Welch et al. (2009)
Recalcitrant chronic rhinosinusitis	Saline nasal irrigation	Squeeze bottle	97%	Yes	Inner surface of the bottle and residual irrigation fluid of the bottle	*CoNS*, *S*. aureus*	*P*. *aeruginosa*, *E*. *cloacae*, Coliforms.	*Candida* spp.	Skin flora	Keen et al. (2010)
Post endoscopic sinus surgery	Saline nasal irrigation	Squeeze bottle		Yes	Irrigation bottle and fluid	CoNS, *S*. *aureus*, *Micrococcus* spp.,	*P*. aeruginosa*, *P*. *mirabilis*, *A*. *calcoaceticus*, *K*. *pneumoniae*, *C*. *freundii*, *S*. *marsescens*		Oropharyngeal flora	Lee et al. (2010)
Chronic rhinosinusitis	Saline nasal irrigation	Squeeze bottle		No	Inner surface and inner tubing of the sinus irrigation bottle	*S*. aureus*, *B*. *anthracis*, *Dietzia* spp., *Paenibacillus* spp., *Brevinibacterium* spp.	*P*. aeruginosa*, *Pseudomonas* spp.*, *Stenotrophomonas* spp., *Acinetobacter* spp., *A*. *tumefaciens*, *Sphingomonas* spp., *Caulobacter* spp., *Afipia* spp., *Erythromicrobium* spp.		Oral flora, skin flora, respiratory flora	Lewenza et al. (2010)
Chronic rhinosinusitis	Saline nasal irrigation	Squeeze bottle with valve		Yes	The nozzle, the inner aspect of the liquid valve	CoNS, *S*. aureus*	*Acinetobacter* spp., *K*. *oxytoca*, *E*. *cloacae*, *C*. *diversus*, *P*. *aeruginosa*, *E*. *coli*.			Foreman and Wormald (2011)
Chronic rhinosinusitis	Nasal steroid spray	Metered dose inhaler spray	72%	Yes	Tip	*CoNS*, *S*. aureus*	*Pseudomonas* spp., *E*. *coli*		Respiratory flora	Tan et al. (2014)
Post endoscopic sinus surgery	Saline nasal irrigation			Yes	Tip and reservoir	*CoNS*, *S*. *aureus*, *Bacillus* spp.	*E*. *coli*, *Acinetobacter* spp., *Enterobacter* spp., *Klebsiella* spp., *Serratia* spp., *Citrobacter* spp., *Flavobacterium* spp., *Pseudomonas* spp.*			Zahedi et al. (2019)
Allergic rhinitis	Saline nasal irrigation	Squeeze bottle	85%	No		Bacilli* and Staphylococci*				Nguyen et al. (2019)

*Most abundant microorganisms; MC, microbial contamination.

Clinical significance:

Concurrent isolated bacteria from the medication device and the nasal cavity of patients with CRS were predominantly *S*. *aureus* (Lee et al., 2010; Keen et al., 2010; Foreman and Wormald, 2011; Foreman et al., 2011). These bacteria in biofilm form are particularly alarming because they are recalcitrant to medical and surgical treatment, and can lead to recurrent sinonasal infection (Keen et al., 2010; Foreman and Wormald, 2011; Foreman et al., 2011; Jervis-Bardy et al., 2011; Tan et al., 2014). Frequently isolated *P*. *aeruginosa* are also known to be potential pathogens causing respiratory infections. These findings highlight the potential risk of contamination in nasal irrigation devices and sprays, which could lead to reintroduction of pathogens into the nasal cavity or sinuses. The presence of biofilms on these devices is particularly difficult to eradicate (Keen et al., 2010; Foreman and Wormald, 2011; Foreman et al., 2011; Jervis-Bardy et al., 2011; Tan et al., 2014). These results emphasize the importance of proper cleaning and maintenance of nasal irrigation devices and sprays, especially for patients with CRS or those who have undergone ESS. The clinical relevance of concurrent microbial contamination of the nasal cavity and medication delivery devices warrants further research, particularly in vulnerable populations, such as immunocompromised patients and those suffering from chronic pulmonary disease, to determine the source and direction of contamination (Welch et al., 2009; Lewenza et al., 2010; Foreman and Wormald, 2011; Foreman et al., 2011; Psaltis et al., 2012; Tan et al., 2014; Nguyen et al., 2019; Zahedi et al., 2019).

### 3.2 Medical devices

#### 3.2.1 Nebulizers

Nebulizer therapy is a mainstay in the management of chronic pulmonary diseases, including cystic fibrosis (CF; Agent and Parrott, 2015; Bell et al., 2020). Device selection is case-based and critical for optimizing clinical efficiency (Agent and Parrott, 2015; Bell et al., 2020), as nebulizer types may vary depending on the patient’s characteristics, drug dosage, and device specifications (Agent and Parrott, 2015; Bell et al., 2020). In certain cases, patients may require the simultaneous use of multiple types of inhalers each with different, sometimes contradictory cleaning instructions (Bell et al., 2020).

The European Respiratory Society and the British Thoracic Society have acknowledged the lack of optimal standardized hygiene instructions for home and hospital-use devices, and stressed the need for a universal code of practice for device maintenance (Bell et al., 2020). Occupational standards such as the Occupational Safety and Health Standard (OSHA) Respiratory Protection Standard (29 CFR 1910.134 Appendix B-2) provide mandatory, detailed cleaning and disinfection procedures for respirators, including temperature limits (maximum 43 °C/110 °F), detergent specifications, and disinfection protocols using hypochlorite or iodine solutions. These evidence-based occupational protocols could inform the development of standardized hygiene instructions for home-use nebulizers. Cleaning and maintenance procedures are suboptimal, particularly among the elderly who face difficulties and require assistance (Boyter and Carter, 2005; Alhaddad et al., 2015). Despite adherence to cleaning, disinfection, and maintenance instructions, nebulizers may be contaminated, predominantly with bacteria, followed by fungi, filamentous yeast, and molds (Alhaddad et al., 2015; Peckham et al., 2016; Riquena et al., 2019). Nebulizer cleaning eliminated Gram-Positive Bacteria (GPB) and fungi, but GNB persisted mainly due to biofilm formation (Jarvis et al., 2014). The presence of biofilm is concerning as it explains the incomplete eradication of GNB even after washing the nebulizer components, such as masks, the device chamber, and mouthpiece (Jarvis et al., 2014). Alarmingly, biofilms, resistant bacteria, and pathogenic strains such as *Burkholderia cepacia*, *S*. *maltophilia*, and *P*. *aeruginosa* have been isolated from contaminated nebulizers (Bell et al., 2020).

The reported microbial contamination rates of home-use nebulizers range from 21 to 93% (Bell et al., 2020; Peckham et al., 2016; Riquena et al., 2019; Tabatabaii et al., 2020; Blau et al., 2007; Yegit et al., 2025), with most studies finding rates between 58 and 75%. The studies span from 2000 to 2025, and the findings remain relatively consistent throughout this period, suggesting that nebulizer contamination has been a persistent issue for CF and Chronic Obstructive Pulmonary Disease (COPD) patients. Limited studies have investigated fungal contamination (Riquena et al., 2019), despite its potential contribution to disease exacerbation and infection (Alhaddad et al., 2015). In fact, nebulizers have been described as reservoirs for pathogens (Hutchinson et al., 1996; Cohen et al., 2006; Jarvis et al., 2014). Frequently isolated GNB include *Pseudomonas* spp. (Barnes et al., 1987; Jones et al., 1985), mainly *P*. *aeruginosa* (Pitchford et al., 1987; Rosenfeld et al., 2001; Cohen et al., 2006; Blau et al., 2007; Jarvis et al., 2014; Tabatabaii et al., 2020), *B*. *cepacia* (Hutchinson et al., 1996), *Acinetobacter* spp. (Barnes et al., 1987; Peckham et al., 2016), *S*. *maltophilia* (Hutchinson et al., 1996), and *Enterobacteriaceae* such as *E*. *coli* (Jarvis et al., 2014), multidrug-resistant (MDR) *S*. *marcescens*, and *Klebsiella* spp. (Barnes et al., 1987; Rosenfeld et al., 2001; Jarvis et al., 2014), and *Flavobacterium* spp. (Barnes et al., 1987). Reported GPB, including *S*. *aureus* (Barnes et al., 1987; Popa et al., 1988; Cohen et al., 2006; Jarvis et al., 2014), *S*. *albus* (Barnes et al., 1987), *Micrococcus* spp. (Barnes et al., 1987), *beta- haemolytic streptococci* (Barnes et al., 1987), *Streptococcus viridans* (Barnes et al., 1987), *diphteroids* (Jones et al., 1985). Limited studies have investigated fungal contamination (Riquena et al., 2019), despite the potential contribution to disease exacerbation and infection (Alhaddad et al., 2015). Some studies reported fungal contamination with *Fusarium oxysporum* (Jarvis et al., 2014), *Aspergillus* spp., in addition to yeasts and other molds (Peckham et al., 2016; Table 3).

**Table 3:** Documented microbial contamination of home-use nebulizers.

Disease	Patient age (years)	Duration of use (Months)	Rate of MC	Contaminated site	Detected microorganisms			Sputum culture	Ref.
					GPB	GNB	Fungi		
CF, Asthma, COPD	1 to 88		46%		*Micrococcus* spp., *S*. *aureus*, *S*. *albus*, *Streptococcus* spp., *S*. *viridans*	*Acinetobacter* spp., *Flavobaterium* spp., *Diphteroids*, *Pseudomonas* spp., *S*. *marcescens*			Barnes et al. (1987)
CF			25%	Compressor tubing, Mouth piece, Other parts		*P*. *putida*, *P*. *fluorescens*, *P*. *testoteroni*, *P*.*s maltophilia*, *P*. *aeruginosa*		*P*. *aeruginosa*	Pitchford et al. (1987)
CF		2.4	69%	Chamber, Compressor tubing, T-piece, Mouth piece	Not assessed	*A*. *johnsonii*, *A*. *junii*, *A*. *radiobacter*, *A*. *xylosoxidans*, *C*. *acidovorans*, *C*. *testosteroni*, *F*. *indologenes*, *F*. *meningosepticum*, *O*. *anthropi*, *O*.*urethralis*, *P*. *aureofaciens*, *P*. *fluorescens*, *P*.*vesicularis*, *S*. *paucimobilis*, *S*. *maltophilia*, *B*. *cepacia*	Not assessed		Hutchinson et al. (1996)
CF	≥7		64%		*S*. *aureus*	*P*. *aeruginosa*, *Haemophilus* spp., *S*. *aureus*	Yeasts unspecified	*P*. *aeruginosa*, *Staphylococcus* spp.	Vassal et al. (2000)
CF	9 months to 44	6 to 12	65%	Mask, mouth piece, reservoir cup, filter	*CoNS*, *M*. *luteus*	*Klebsiella* spp., *Acinetobacte*r spp., *Enterobcater* spp., *Proteus* spp., *Pseudomonas* spp., *P*. *aeruginosa*, *E*. *coli*, *M*. *luteus*, *CoNS*	*C*. *albicans*	*Aspergillus* spp., *C*. *albicans*, *S*. *aureus*, *P*. *aeruginosa*	Blau et al. (2007)
CF	1 to 7		21%	Chamber, mask, mouth piece	*Bacillus* spp., *CoNS*., *S*. *aureus*	*A*. *xylosoxidans*, *K*. *pneumoniae*, *K*. *ozaenae*, *P*. *aeruginosa*, *S*. *maltophilia*, *S*.*marcescens*	Yeasts (unspecified)	*S*. *aureus*, *S*. *aureus Methicillin resistant*, *B*. *cepacia*, *P*. *aeruginosa*, *Mucoid P*. *aeruginosa*	Brzezinski et al. (2011)
CF	11.2 ± 3.74		58%	Mouth piece, reservoir cup	*CoNS*, *S*. *aureus*, *Gram-positive bacilli*	*Ac*. *xylosoxidans*, *Klebsiella* spp., *P*. *aeruginosa*, *P*. *putida*, *S*.*maltophilia*, *Acinetobacter* spp., *B*. *cepacia*, *E*. *coli*, *Enterobacter* spp., *Non-fermenting gram-negative bacilli*, *P*. *fluorescens*, *Non-fermenting gram-negative bacilli*	Yeasts (unspecified)		Zuana et al. (2014)
COPD	40 to 93		73%	Chamber, compressor tubing, mask, mouth piece	Bacillus spp., *S*. *aureus*, CoNS	Enterobacteriaceae, *K*. *pneumoniae*, *P*. *aeruginosa*, *E*. *coli*, Diphteroids, multidtug -resistant *S*. *marcescens*, multidrug-resistant coliforms	*F*. *oxysporum*, *Candida* spp.		Jarvis et al. (2014)
CF			58%		Not assessed	Not assessed	*A*. *fumigatus*, *A*.*s niger*, *A*. *versicolor*, *C*. *sphaerospermum*, *E*. *oligosperumum*, *E*. spp., *E*. *jeanselmei*, *L*. *lecanii*, *L*.sp., *M*. *fulvum*, *M*. spp., *P*. *commune*, *P*. *glabrum*, *P*. *griseofulvin*, *P*. *coryphilum*, *P*.*digitatum*, *Penicillium* sp., *R*. *oryzae*, *S*. *chartarum*, *U*. *chartarum*, *A*. *pullulans*, *C*. *albicans*, *C*. *guilliermondii*, *C*. *holmii*, *C*. *krusei*, *C*. *lipolytica*, *C*. *parapsilosis*, *C*. *pelliculosa*, *C*. *sake*, *C*. spp., *C*. *zeylanoides*, *C*. spp., *C*. *albidus*, *C*. *unigutatulus*, *C*. *carnescens*, *R*. *glutinis*, *R*. *mucilaginosa*, *R*. *minuta*, *Rhodotorula* sp., *S*. *roseus*, *T*. *asahii*	*A*. *fumigatus*, *A*. *niger*, *A*. *versicolor*, *C*. *sphaerospermum*, *Exophiala* sp., *Penicillium* sp., *C*. *albicans*, *C*. *parapsilosis*, *Candida* sp., *R*. *glutinis*, *R*.*mucilaginosa*	Peckham et al. (2016)
CF	16.99 ± 11.24		93%	Compressor tubing, mask, mouth piece	*Corynebacterium* spp., *S*. *pasteuri*, *S*. *warneri*, *Streptococci*	*Moraxella* spp., *P*. *stuzeri*		*As flavus*, *A*. *niger*, *C*. *tropica*, *C*. *albicans*, *P*. *aeruginosa*, *S*. *marcescens*, *S*. *aureus*	Manor et al. (2017)
CF	15.8 ± 6.5		72%	Reservoir cup, interface	*Bacillus* spp., *Staphylococcus* spp., *S*. *aureus*, Oxacillin-resistant coagulase-negative *S*. *aureus*, *Micrococcus* spp., *Streptococcus* spp.,	*Acinetobacter* spp., *Delftia* spp., *Klebsiella* spp., *Pseudomonas* spp., Mucoid and Non-mucoid *P*. *aeruginosa*, *Enterobcater* spp., *S*. *marcescens*, *M*. *osloensis*, *A*. *hydrophila*, *C*. *indologenes*, *C*. *testosteroni*, *Stenotrophomonas* spp., *S*. *maltophilia*, *Burkholderia* spp., *B*. *cepacia complex*, *Sphingobacterium* spp., *S*. *paucimobilis*, *P*. *agglomerans*, *R*. *radiobacter*	*A*. *niger*, *Penicillium* sp., *C*. *albicans*, *Candida* spp., *Non-albicans Candida* spp., *Rhodotorula* spp., *Cladosporium* spp.	Not assessed	Riquena et al. (2019)
CF	6 to 18		75%	Chamber, mouth piece	B*acillus* spp., *S*. *epidermidis*, *S*. *aureus*, *S*. *saprophyticus*, *A*. *viscosus*, *E*. *faecalis*, *G*.*adiacens*, *Microbacterium* spp., *Micrococcus* spp., *M*. *luteus*, *Paenibacillus* spp., *Rothia* spp., *Viridans group* *Streptococcus*	*E*. *coli*, *A*. *junii*, *A*. *radioresistens*, *H*. *parahaemolyticus*, *N*. *subflava*, *R*. *mucilaginosa*,	*C*. *albicans*	*Oral flora*, *S*. *aureus*, *P*. *aeruginosa*, *Aspergillus* spp. *and S*. *maltophilia*	Murray et al. (2019)
CF	7.6 ± 4.2	42	70.50%	Mask, mouth piece, reservoir cup	*CoNS*, *S*. *aureus*, *Micrococcus* spp.	*Enterobcater* spp., *Pseudomonas* spp., *P*. *aeruginosa*.	*C*. *albicans*	*CoNS*, *Pseudomonas* spp., *Enterobacter* spp., *Micrococcus* spp., *S*. *aureus*	Pitchford et al. (1987)

MC, Microbial contamination; CF, Cystic fibrosis; COPD, Chronic obstructive pulmonary disease.

Clinical significance:

Contaminated nebulizers can act as reservoirs for pathogens (Kernen et al., 2005; Saiman and Siegel, 2004; Weber et al., 2014; Reychler et al., 2009; Tai et al., 2011; Wexler et al., 1991; Cobben et al., 1996), particularly GNB such as *P*. *aeruginosa*, leading to transmission, colonization, and disease exacerbation in patients, including those with cystic fibrosis and COPD (Pitchford et al., 1987; Hutchinson et al., 1996; Kernen et al., 2005; Cobben et al., 1996). Microbial transfer can occur bi-directionally, either from patient secretions to the device or from a contaminated device to the patient (Saiman and Siegel, 2004; Manor et al., 2017). Persistent colonization with *P*. *aeruginosa* in CF patients is a major cause of pulmonary deterioration and death (Bell et al., 2020). Nebulizers are also potentially contaminated by the storage environment (Jones et al., 1985; Hutchinson et al., 1996). Studies suggest nebulizers can be used for up to 72 h without heavy microbial contamination (Weber et al., 2014; Bell et al., 2020). Of note, some studies reported poor to no correlation between the microbial contamination of the device and sputum samples despite inadequate cleaning (Rosenfeld et al., 2001; Brzezinski et al., 2011).

#### 3.2.2 Breast pumps

Human milk contributes to the normal development and establishment of microbial microflora in infants (Serra et al., 2013; Toscano et al., 2017). The breast milk commensal bacterial flora is dense and diverse, including *Staphylococcus* spp., *Lactobacillus* spp., *Enterococcus* spp., *Propionibacterium* spp., and *Corynebacterium* spp. (Marín et al., 2004; Serra et al., 2013).

Human milk influences initial intestinal microbiota and modulates the newborn’s immune system (Toscano et al., 2017). The acceptable microbial load of expressed milk is variable, but it should be less than 10^5^ CFU/mL for mesophilic aerobic bacteria and 10 CFU/mL for enterobacteria (Serra et al., 2013). Expression of milk at home using breast pumps offers multiple advantages to the mother and infant, but can also be problematic to both (Boo et al., 2001; Rasmussen and Geraghty, 2011; Serra et al., 2013). One of the critical problems associated with the use of these devices is the risk of milk microbial contamination during transfer, or as a result of inadequate cleaning and disinfection (Moloney et al., 1987; Boo et al., 2001; Brown et al., 2005; Voelz et al., 2010; Rasmussen and Geraghty, 2011; Faro et al., 2011; Labiner-Wolfe and Fein, 2013; Felice et al., 2017a; Felice et al., 2017b). Breast pump contamination with high levels of fungi, yeasts, and potential pathogenic bacteria, such as *E*. *coli*, *S*. *aureus*, *Enterococcus faecalis*, *Pseudomonas* spp., *Proteus* spp., and *Salmonella* spp., is unacceptable and indicates non-hygienic conditions (Serra et al., 2013). A randomized controlled study showed that participants struggle to clean the collection kits tiny cracks because they are difficult to reach (Paredes, 2019). The breast pump valves through which milk passes from the flange into the collection bottle contain debris and build-ups from milk and fat, serving as a bacterial reservoir for growth (Paredes, 2019). Data showed bacterial growth, including GNB, such as *S*. *marcescens*, *Klebsiella* spp., and *E*. *coli* (Moloney et al., 1987; Gransden et al., 1986; Voelz et al., 2010; Faro et al., 2011), *P*. *aeruginosa*, and *Acinetobacter* spp. (Moloney et al., 1987; Voelz et al., 2010; Faro et al., 2011; Engür et al., 2014; Reyes et al., 2025; Peters et al., 2016), *Stenotrophomonas* spp. (Reyes et al., 2025), and GPB such as *S*. *aureus*, *E*. *faecalis* (Moloney et al., 1987; Voelz et al., 2010; Faro et al., 2011; Peters et al., 2016), and *Clostridium perfringens* (Liu et al., 2023). Serra et al. (2013) found that more than half of the home-expressed breast milk (59.6%) using a pump contained higher bacterial count than expressed milk stored in healthcare settings (39.6%; Serra et al., 2013). Home samples harbored more than 10^5^ CFU/mL mesophilic aerobic bacteria, and some samples showed growth of yeasts, fungi, and other pathogenic bacteria (Serra et al., 2013). The authors considered that microbial contamination indicates inadequate breast pump cleaning, hand washing, transportation conditions, pumping practices, or failure to follow instructions, especially in the absence of professional supervision at home (Geraghty, 2011; Mense et al., 2013; Serra et al., 2013; Price et al., 2016; Felice et al., 2017a; Felice et al., 2017b; Froh et al., 2018). Another study showed that differences in in-use pumping supplies influence the milk microbiome (Reyes et al., 2025).

Clinical significance:

While the clinical implications of microbial contamination in expressed breast milk remain unclear for healthy term infants (Serra et al., 2013; Schanler et al., 2011), proper hygiene during pumping is imperative, especially for vulnerable premature neonates. The Centers for Disease Control and Prevention (CDC) has issued advisories stressing meticulous cleaning and sanitization of breast pump equipment following a tragic case of a premature infant succumbing to *Cronobacter sakazakii* meningitis linked to contaminated pump parts (Haston, 2023). This bacterium poses severe risks to susceptible infants under 2 months old, preterm, or immunocompromised. Studies have reported breast milk contamination with various pathogens like *S*. *aureus*, *S*. *epidermidis*, and *Enterobacter* spp. (Gad et al., 2021) and *S*. *marcescens* associated with neonatal gastrointestinal distress (Navadifar et al., 2023).

A study including 393 mothers from the CHILD (Canadian Healthy Infant Longitudinal Development) birth cohort study raised concerns about the implications of pump-related microbial contamination in altering breast milk microbiota (Moossavi et al., 2019). Their analysis demonstrated enrichment of taxa and potential opportunistic pathogens such as *Stenotrophomonas* spp. in milk from indirect breastfeeding via pumps, suggesting derivation from environmental sources (Moossavi et al., 2019). The authors hypothesized that this enrichment of potential pathogens increases the risk of respiratory infections and asthma, given their prior findings linking pumped milk to asthma incidence. However, they acknowledged the need for further research to elucidate the mechanisms by which microbiota alterations from pumping may impact infant health and development (Moossavi et al., 2019). While the direct clinical repercussions remain to be fully elucidated, emerging evidence highlights the importance of rigorously maintaining pump hygiene, especially for premature and immunocompromised neonates, to mitigate potential risks from breast milk contamination and microbiota dysbiosis induced by pumping practices.

### 3.3 Child-care products

#### 3.3.1 Feeding bottles

Microbial contamination of feeding bottles, teats, and food content is a public health concern, predominantly in developing countries (Elegbe et al., 1982; Cherian and Lawande, 1985; Suthienkul et al., 1999; Andresen et al., 2007; Gibson et al., 2017; Rothstein et al., 2019; Bick et al., 2020; Marege et al., 2023). Enteric bacteria are found in the feeding bottle contents, on and in the inner teat surfaces and screw cap (Elegbe et al., 1982; Cherian and Lawande, 1985; Tesfaye, 1992; Suthienkul et al., 1999; Andresen et al., 2007; Redmond et al., 2009; Gibson et al., 2017; Rothstein et al., 2019; Ayaz et al., 2020; Marege et al., 2023). Studies showed that the predominant microbial contaminant of interest is *E*. *coli* (Surjono et al., 1980; Elegbe et al., 1982; Cherian and Lawande, 1985; Tesfaye, 1992; Suthienkul et al., 1999; Andresen et al., 2007; Redmond et al., 2009; Gibson et al., 2017; Rothstein et al., 2019; Ayaz et al., 2020; Marege et al., 2023). Other enteric bacteria were isolated from the bottle surface and teats, including *Citrobacter* spp. (Tesfaye, 1992), *Enterobacter* spp. (Suthienkul et al., 1999; Rachon et al., 2017), *Klebsiella* spp. (Suthienkul et al., 1999; Cherian and Lawande, 1985; Tesfaye, 1992), *S*. *marcescens* (Rachon et al., 2017), and non-coliform bacteria such as *Salmonella paratyphi* (Cherian and Lawande, 1985) and *Shigella* spp. (Tesfaye, 1992). Additional reported GNB on the bottle surface, and the teats were *Aeromonas* spp. (Suthienkul et al., 1999), including *Aeromonas hydrophila* (Cherian and Lawande, 1985; Ayaz et al., 2020) and *Vibrio cholerae* non-O1 (Suthienkul et al., 1999). Isolated GPB showed predominance of *S*. *aureus*, *Bacillus* spp. (Cherian and Lawande, 1985; Ayaz et al., 2020), and *E*. *faecalis* (Cherian and Lawande, 1985). *C*. *albicans* was also isolated from the feeding bottles and teats (Cherian and Lawande, 1985). In particular, biofilm formation is of great concern because of its resistance to regular cleaning practices (Rachon et al., 2017; Rothstein et al., 2019). Microbial contamination of home-prepared bottles is affected by socioeconomic determinants, such as the educational level and hygiene practices, low-economic settings and water quality, lack of awareness and education of new mothers that follow old family methods and advise for cleaning and disinfecting feeding bottles, use of powdered milk formula machines (Rothstein et al., 2019) that may not reach target water temperature > 70 °C, necessary for killing the main part of microorganisms (Imong et al., 1995; Gibson et al., 2017; Rachon et al., 2017; Rothstein et al., 2019; Ayaz et al., 2020; Marege et al., 2023). The variability of current methods of disinfection can also influence the microbial load in feeding bottles (Redmond et al., 2009). A study comparing different disinfection methods reported that *S*. *aureus* and other microbial contaminants were isolated from bottles disinfected with an electric steamer, while microwave use and cold disinfection methods showed better results (Redmond et al., 2009). Even if the disinfection method is adequate, extrinsic factors such as hand contact, dirty preparation surface, or use of a dishwasher that can itself be a source of contamination can be involved (Redmond et al., 2009).

#### 3.3.2 Pacifiers

Latex rubber and silicone Pacifiers (dummies) can be a source of microbial contamination, including gram-negative bacilli, gram-positive cocci, biofilms, yeasts, and molds (Niemelä et al., 1995; Ollila et al., 1998; Fleming and Golding, 2000; Briggs, 2006; Silveira et al., 2009; Bullard et al., 2012; de Coimbra Paula et al., 2020). Comina et al. (2006) showed that latex rubber dummies are more prone to contamination and biofilm formation than silicone-based ones (Comina et al., 2006). Pacifiers can harbor *Candida* spp. (Silveira et al., 2009; Lopes et al., 2019), and non-pathogenic bacteria, such as *lactobacilli* (Sio et al., 1987; Ollila et al., 1997; Mattos-Graner et al., 2001). Pacifiers can be contaminated with pathogenic bacteria, such as *S*. *aureus* and *K*. *pneumoniae*, *Streptococcus* spp., *Enterococcus* spp., and molds (Pedroso et al., 2018; de Coimbra Paula et al., 2020). de Coimbra Paula et al. (2020) conducted a structural and microbiological analysis of silicone-based pacifiers and found microbial growth of *CoNS*, *S*. *aureus*, *Streptococcus* spp., *P*. *aeruginosa*, *Bacillus* spp., *Klebsiella pneumoniae*, *Citrobacter freundii*, and *Candida* spp. (de Coimbra Paula et al., 2020). Pacifiers should be replaced and continuously cleaned and disinfected (Souza et al., 2020). The variable cleaning and disinfecting methods have different levels of effectiveness (Chamele et al., 2012; Nelson-Filho et al., 2015). Nelson-Filho et al. (2015) showed that dummies cleaned with tap water harbor *Streptococcus mutans*, a bacterium associated with dental caries in humans (Nelson-Filho et al., 2015).

Clinical significance:

At birth, the immune system is not fully developed, which can enhance their susceptibility to infections. Infants receive early protection against infectious diseases through the passive transfer of IgG antibodies from the mother via transplacental routes during birth and through breast milk during breastfeeding (Simon et al., 2015; Langel et al., 2022). As the innate and adaptive immune systems mature over time, the child may become less vulnerable to infections (Simon et al., 2015; Langel et al., 2022). However, during this developmental process, exposure of a child under 5 years of age to bacteria, particularly those forming biofilms, is concerning. Exposure to microbial contaminants from child-care devices is a risk for the occurrence of otitis media (Niemelä et al., 1995; Salah et al., 2013; Nelson-Filho et al., 2015; Souza et al., 2020), dental caries, and various infections (Ollila et al., 1998; Vázquez-Nava et al., 2008), as well as intestinal parasitic infections (Comina et al., 2006; Nelson-Filho et al., 2015). Consequently, stringent hygiene practices and preventive measures are imperative to safeguard the health and well-being of this vulnerable population.

### 3.4 Cosmetic products

In household settings, the hazardous consumer behavior can enhance the risk of microbial contamination (Welch et al., 2009). These behaviors include inadequate storage conditions, such as in the bathroom (Eldesoukey et al., 2016), dropping the product such as a beauty brush on the floor and using it without cleaning, using cosmetics beyond the expiry date (Giacomel et al., 2013; Skowron et al., 2017), sharing items (Skowron et al., 2017), putting water or saliva on them, not cleaning items, where applicable, and inadequate hand hygiene before cosmetics application (Food and Drug Administration, 2024). Home-use cosmetic products, including lipsticks, lip gloss, foundation, mascara, eyeliner, eye shadows, beauty blenders, and al-kohl, were tested after consumer use to investigate potential microbial contamination (Wilson et al., 1975; Abdelaziz and Alkofahi, 1989; Abdelaziz et al., 1989; Pack et al., 2008; Ravita et al., 2009; Onurdağ et al., 2010; Saeed and Asif, 2011; Giacomel et al., 2013; Eldesoukey et al., 2016; Skowron et al., 2017; Siya et al., 2019; Table 4).

**Table 4:** Documented microbial contamination of home-use cosmetics.

Cosmetics	Rate of MC	Detected microorganisms			Ref.
		GPB	GNB	Fungi	
Mascara		*Arthrobacter roseus*, *Bacillus* spp., *Corynebacterium* spp., *Diphteroids*, *Micrococcus* spp., *Staphylococcus* spp., *Streptococcus* spp., *Lactobacillus* spp.	*K*. *pneumoniae*, *P*. *aeruginosa*	*Candida* spp., *C*. *albicans*, *C*. *parapsilosis*, *Penicillium*, *Fusarium*, *Aspergillus*, *Trichoderma*, *Cephalosporium*, *Molds*	Wilson et al. (1975)
Al Khol	>85%	*Bacillus* spp., *Staphylococcus* spp., *S*. *aureus*	*Proteus vulgaris*, *Pseudomonas* spp., *P*. *aeruginosa*, *S*. *marscesens*	*unspecified*	Abdelaziz and Alkofahi (1989)
Mascara	36%	*S*. epidermidis*, *Streptococcus* spp.		*Detected fungi unspecified*	Pack et al. (2008)
Mascara, Non-specified face and eye products	-	*Bacillus* spp., *Corynebacterium* spp., *Staphylococcus* spp., Staphylococcus aureus*, *Streptococcus* spp., *Lactobacillus* spp.	*Pseudomonas* spp.	*Yeasts*	Ravita et al. (2009)
Eye shadows, Eye lashes, foundation, Lipstick	14%	*Bacillus* spp., *S*. *aureus*, *S*. *epidermidis*, *Streptococcus* spp.	*E*. *coli*	*C*. *albicans*	Onurdağ et al. (2010)
Lipstick	31%	*Bacillus* spp. *, *saprophyticus*, *S*. *aureus*, *S*. *epidermidis*, *Streptococcus* spp., *M*. *sedentarius*			Saeed and Asif (2011)
Mascara	100%	*S*. aureus*	*P*. *aeruginosa*		Giacomel et al. (2013)
Lipstick	93%	*S*. *saprophyticus*, *S*. *aureus*, *S*. epidermidis*, *Streptococcus* spp.			Siya et al. (2019)
Lipstick	70–90%		*C*. *freundii*, *P*. *monteilii*, *P*.*fulva*	*-*	Bashir and Lambert (2020)
Mascara	70–90%	*S*. *saprophyticus*	*P*. *gergoviae*	*-*	
Beauty blenders	70–90%		*P*. *gergoviae*, *C*. *freundii*, *E*. *coli*, *P*. *aeruginosa*, *P*. *monteilii*		
Lipgloss	70–90%	*B*. *litoralis*, *M*. *luteus*, *S*. *haemolyticus*, *S*. *cohnii*, *S*. *capitis*, *S*. *pasteurii*, *Lactobacillus* spp.	*C*. *freundii*, *P*. *monteilii*, *P*.*fulva*, *P*. *putida*	*C*. *glabrata*	
Eyeliner	70–90%	*Arthrobacter roseus*, *Bacillus muralis*, *Cryptococcus diffluens*, *S*. *haemolyticus*, *S*. *saprophyticus*, *S*. *cohnii*, *S*. *capitis*	*Burkholderia vietnamiensis*, *E*. *coli*		

*Most abundant microorganisms.

#### 3.4.1 Mascara

The contamination of used mascara with various pathogenic and non-pathogenic microorganisms raises health concerns, particularly in vulnerable individuals. The detection of *S*. *aureus* (Wilson et al., 1975; Ravita et al., 2009; Giacomel et al., 2013) and *P*. *aeruginosa* (Wilson et al., 1975; Giacomel et al., 2013) in used mascara raises the risk of skin and eye infections, particularly if the mascara comes into contact with mucous membranes or broken skin (Taylor and Unakal, 2017; O’Callaghan, 2018). *S*. *aureus* is a known human pathogen that can cause serious infections, including skin and soft tissue infections such as impetigo, folliculitis, cellulitis, and scalded skin syndrome (Taylor and Unakal, 2017; Astley et al., 2023). *S*. *aureus* is a serious ophthalmic pathogen that can infect the ocular adnexa, such as the cornea, leading to keratitis or the inner chambers of the eye, causing endophthalmitis in susceptible individuals. These two types of infections often lead to the loss of visual acuity or even blindness (Tong et al., 2015). The risk of ocular infections increases in cases of contact lens wear, recent ocular surgery or trauma, intravitreal injection, preexisting ophthalmic diseases, the long-term use of topical or systemic steroids, and immunosuppressants (Iskandar et al., 2022). Additional ocular diseases associated with *S*. *aureus* include blepharitis, dacryocystitis, and conjunctivitis (Tong et al., 2015). Other detected microorganisms known to cause ocular diseases, ranging from conjunctivitis, keratitis, and endophthalmitis (Astley et al., 2023), included *S*. *epidermidis* (Wilson et al., 1975; Pack et al., 2008), *Streptococcus* spp. (Wilson et al., 1975; Pack et al., 2008; Siya et al., 2019), *Micrococcus* spp. (Wilson et al., 1975), *Bacillus* spp. (Wilson et al., 1975; Ravita et al., 2009), *Corynebacterium* spp. (Wilson et al., 1975; Ravita et al., 2009), and other *diphteroids* (Wilson et al., 1975). Detected Lactobacilli may rarely cause bacteremia in immunocompromised patients (Astley et al., 2023; Kullar et al., 2023).

Isolated *P*. *aeruginosa* (Wilson et al., 1975; Giacomel et al., 2013) and *C*. *albicans* (Wilson et al., 1975) *a*re known causes of opportunistic infections and life-threatening acute and chronic diseases, particularly in immunocompromised individuals (Hassan et al., 2010; Moradali et al., 2017; Hemaid et al., 2021). *P*. *aeruginosa* can cause acute conjunctivitis, contact-lens-associated keratitis, endophthalmitis, and dacryocystitis (Astley et al., 2023; Lin et al., 2022). Other detected GNB ocular pathogens (Astley et al., 2023) were *K*. *pneumoniae* (Wilson et al., 1975), *Pluralibacter Gergovia* (Furlan and Stehling, 2023) also known as *Enterobacter gergoviae*, isolated from mascara, is a multidrug-resistant species that can cause opportunistic infections and even outbreaks (Furlan and Stehling, 2023). Detected fungi in tested in-use mascara, including yeasts such as *C*. *albicans* (Wilson et al., 1975), and filamentous forms, such as *Fusarium* spp. (Wilson et al., 1975), *Aspergillus* spp. (Wilson et al., 1975), *Penicillium* spp. (Wilson et al., 1975), *Trichoderma* spp. (Wilson et al., 1975), and *Cephalosporium* spp. (Wilson et al., 1975) can cause ocular infections (Petrillo et al., 2023). Keratitis due to *Candida* spp., including *C*. *parapsilosis*, are more commonly encountered in patients with chronic ocular surface diseases and systemic diseases (Bourcier et al., 2017; Petrillo et al., 2023). The filamentous forms can more frequently infect individuals who wear contact lenses (Bourcier et al., 2017; Petrillo et al., 2023). *Candida* spp. including *C*. *albicans* and *C*. *parapsilosis*, are also known to cause choroiditis in immunocompromised individuals, drug addicts, people on corticosteroids, and parenteral or broad-spectrum antibiotic treatment of septicemia (Bourcier et al., 2017). Endophthalmitis due to fungal infection is mainly seen in immunocompromised individuals, drug addicts, and patients using corticosteroids or on broad-spectrum antibiotic treatment of septicemia (Sheu, 2017).

#### 3.4.2 Al-kohl, eye liners, and eye shadows

The tested eyeliners were contaminated with other *Staphylococcus* spp. including *S*. *saprophyticus* (Bashir and Lambert, 2020), *S*. *haemolyticus*, *S*. *hominis*, *S*. *capitis*, in addition to *Micrococcus luteus*, *Bacillus muralis*, and *Arthrobacter roseus* (Bashir and Lambert, 2020). GNB included *Burkholderia vietnamiensis and E*. *coli* (Bashir and Lambert, 2020). Other studies showed the presence of yeast-like fungi *Cryptococcus diffluens*, and bacteria, such *as Arthrobacter roseus* and *B*. *vietnamiensis*, indicating mainly an environmental exposure of the product. *B*. *vietnamiensis* is a documented health risk for immunocompromised individuals (Bashir and Lambert, 2020). On the other hand, al-khol was contaminated with *S*. *aureus*, *P*. *aeruginosa*, and unspecified fungi (Abdelaziz and Alkofahi, 1989). They also exhibited growth of *Bacillus* spp., *Pseudomonas* spp., and *S*. *marcescens* (Abdelaziz and Alkofahi, 1989). In-use tested eye shadows showed microbial growth of *S*. *aureus* and *C*. *albicans* (Onurdağ et al., 2010). Additional detected microorganisms were *S*. *epidermidis*, *Bacillus* spp., *Streptococcus* spp., and *E*. *coli* (Onurdağ et al., 2010).

#### 3.4.3 Lip gloss and lipsticks

Home-used lipsticks and lip gloss were contaminated with numerous GPB, GNB, and fungi. Exposure to these microorganisms can lead to infections occurring through direct contact between the contaminated item and the skin, lips, and mucous membranes around the lips or through small cuts, wounds, and cracks on the lips and surrounding skin. Microbial contaminants can be ingested into the digestive system during eating or drinking, leading to gastrointestinal infections, particularly in immunocompromised individuals. The presence of *S*. *aureus* (Saeed and Asif, 2011; Siya et al., 2019), *P*. *aeruginosa* (Giacomel et al., 2013), and *C*. *albicans* (Onurdağ et al., 2010) is of particular concern.

*S*. *aureus* can cause infection of the tissue around the lips, including impetigo (Del Giudice, 2020), cheilitis and cellulitis (Saraux et al., 2023), folliculitis (Saraux et al., 2023). *S*. *aureus* disseminates from the oral cavity to the gut and other body sites, causing serious systemic diseases (Bruno et al., 2007; Ohara-Nemoto et al., 2008; Lucerna et al., 2015; McCormack et al., 2015; Zawadzki et al., 2016; Kitamoto et al., 2020; Del Giudice, 2020; Aleem et al., 2020; Amin et al., 2021; Raineri et al., 2022; Esmkhani and Shams, 2022; Jabeen et al., 2023; Santacroce et al., 2023), such as pneumonia (Bruno et al., 2007), particularly in immunocompromised patients, including those with Human Immunodeficiency Virus (HIV; Aleem et al., 2020). GPB isolated from lipstick and lip gloss are part of the normal microbiota on the skin and mucous membranes, but their presence in high quantities can lead to potential health hazards, including skin infections, oral and gastrointestinal infections. The isolated bacteria GPB from home-use lip gloss (Ravita et al., 2009; Saeed and Asif, 2011; Giacomel et al., 2013; Siya et al., 2019; Bashir and Lambert, 2020) included *S*. *epidermidis*, *S*. *saprophyticus*, *S*. *haemolyticus*, *S*. *cohnii*, *S*. *capitis*, *S*. *pasteurii* in addition to *Micrococcus* spp., such as *Micrococcus luteus*, and *Micrococcus sedentarius* and other GPB including *Streptococcus* spp., *Lactobacillus* spp., *Bacillus* spp., such as *B*. *litoralis*. *Staphylococcus* spp. including *S*. *aureus*, *S*. *epidermidis*, *S*. *haemolyticus*, and *S*. *capitis* may be linked to infective endocarditis generated from the oral route (Siya et al., 2019). *Streptococcus* spp. are typical GPB of the oral cavity. Depending on the species, they may be the leading cause of plaque formation, oral infection such as tonsillopharyngitis, extra-oral infections, including otitis media and pneumonia (Santacroce et al., 2023). Under specific conditions, certain *Streptococcus* spp. may cause bacteremia following tooth extraction and even endocarditis (Santacroce et al., 2023). The transmission from mouth to gut is being studied as a main driver of gastrointestinal tract infections (Kitamoto et al., 2020). Detected *Bacillus* spp. in lip gloss and lipstick (Saeed and Asif, 2011; Bashir and Lambert, 2020) are known causes of cutaneous, infections (Esmkhani and Shams, 2022).

GNB includes *Pseudomonas* spp. (Abdelaziz et al., 1989; Ravita et al., 2009; Bashir and Lambert, 2020), *P*. *monteilii* (Giacomel et al., 2013; Bashir and Lambert, 2020), *P*. *fulva* (Bashir and Lambert, 2020), *P*. *putida* (Bashir and Lambert, 2020), in addition to *E*. *coli* (Bashir and Lambert, 2020; Onurdağ et al., 2010), and *C*. *freundii* (Bashir and Lambert, 2020). *C*. *freundii* in particular is becoming an increasing public health concern and is the leading cause of gastrointestinal infections, urinary tract infections, and bacteremia (Elmorsy and Hafez, 2016), particularly in patients with underlying medical conditions such as cardiovascular and renal diseases, leukemia, diabetes, neurologic diseases, and urinary tract deformities (Jabeen et al., 2023). *E*. *coli* may also cause respiratory, gastrointestinal, and urinary tract infections, particularly in individuals with weakened immune systems (Zawadzki et al., 2016). *P*. *aeruginosa* can cause infections with various severity, particularly in patients with CF, diabetes, and other immunocompromised patients (Raineri et al., 2022). Infections include skin and soft tissues, such as pseudomonal folliculitis, in addition to other life-threatening conditions such as endocarditis and meningitis, sepsis, and septic shock (Raineri et al., 2022).

*C*. *albicans* and, less commonly, *C*. *glabrata*, may be found in contaminated lip gloss (Bashir and Lambert, 2020). It can contribute to oral candidiasis in both immunocompetent and vulnerable individuals (Taylor et al., 2019). *C*. *albicans* is also the leading cause of angular cheilitis, an inflammatory skin condition located at the labial commissure (Federico et al., 2019). Elderly people are particularly susceptible to these types of infections (Santacroce et al., 2023).

#### 3.4.4 Foundation and beauty blenders

The foundation showed microbial growth of *S*. *aureus* and *C*. *albicans* (Onurdağ et al., 2010). Additional detected microorganisms were *S*. *epidermidis*, *Bacillus* spp., *Streptococcus* spp., *and E*. *coli* (Onurdağ et al., 2010). Beauty blenders were contaminated with GNB, including *Acinetobacter ursingii*, *P*. *monteilii*, and *E*. *coli* (Bashir and Lambert, 2020). *S*. *aureus*, *Streptococcus* spp., *Bacillus* spp., and *E*. *coli* are known causes of skin and soft tissue infections, in addition to other systemic diseases in susceptible individuals. Unlike commercially manufactured cosmetics with applied quality control, preservative optimization, and microbiological testing, homemade preparations lack standardized formulation protocols and adequate preservation systems. The absence of proper preservatives, combined with non-sterile preparation conditions, inadequate storage, and the use of natural ingredients prone to microbial growth (e.g., oils, butters, and botanical extracts), creates an ideal environment for bacterial and fungal contamination (Couteau et al., 2022). Furthermore, individuals preparing these products at home often lack knowledge of good manufacturing practices, proper sanitation techniques, and appropriate container sterilization methods. More recently, ultrasonic water baths marketed for cleaning makeup brushes have recently entered the consumer market, though peer-reviewed evidence supporting their antimicrobial efficacy in home settings is currently lacking. The risks are compounded by the complete absence of post-production microbiological monitoring and shelf-life stability testing. Of particular concern is the lack of regulatory oversight and surveillance data on homemade cosmetic-related infections, making it impossible to assess the true public health impact of this practice. This data gap prevents adequate risk assessment and the development of evidence-based safety guidelines for consumers who choose to prepare their own cosmetic products.

##### 3.4.4.1 Contact lens case

The contact lens case (CLC) is the most frequently contaminated lens care item with bacteria, fungi, and protozoa (Donzis et al., 1988; Boost and Cho, 2005; Yung et al., 2007a; Yung et al., 2007b; Thakur and Gaikwad, 2014). Microbial contamination of CLC varies between 19 and 92% regardless of the cleaning care solution (Mayo et al., 1986; Donzis et al., 1988; Wilson et al., 1990; Larkin et al., 1990; Simmons et al., 1991; Fleiszig and Efron, 1992; Devonshire et al., 1993; Gray et al., 1995; Midelfart et al., 1996; Pens et al., 2008; Willcox et al., 2010; Kratz et al., 2011; Kuzman et al., 2014; Dantam et al., 2016; Eslami et al., 2020). The discrepancies in the reported levels of microbial contamination may be due to the differences in sampling site, such as well, upper and lower CLC rim, infrequent or lack of CLC regular replacement, mismatched lens solutions and CLC, patient compliance to instructions and inappropriate lens care behaviors, gender differences, hygiene factors, intended lens wear use such as cosmetic or therapeutic purposes, CLC design, and material, duration of use, different users such as asymptomatic versus experienced wearers (Yung et al., 2007a; Yung et al., 2007b; Wu et al., 2010; Morgan et al., 2011; Eslami et al., 2020).

The CLC sampling site is often unspecified across different studies (Wu et al., 2010). Wu et al. (2010) found a difference in microbial contamination between the CLC upper and lower inner rims (Wu et al., 2010). Kuzman et al. (2014) found 23% microbial contamination inside the CLC vs. 39% on the rim. The author considered that the CLC rim was not in long-term contact with the disinfectant solution (Wu et al., 2010). CLC should be replaced every 3 months, as recommended, to avoid microbial colonization (Wu et al., 2010; Morgan et al., 2011; Cope et al., 2017; Eslami et al., 2020; Waghmare and Jeria, 2022). Eslami et al. (2020) found that a duration of CLC use of less than 3 months was associated with a lower level of contamination (2.6%) than 9 months or more (48.4%; Eslami et al., 2020). Wu et al. (2015) confirmed this finding, while other studies reported that regular replacement of CLC did not contribute to any improvement in microbial contamination (Yung et al., 2007a; Yung et al., 2007b). Microbial contamination of CL can occur within 1 week of use, and lens bioburden usually increases within 2 weeks (Lakkis et al., 2009) The clinical implications of the association between regular replacement and levels of microbial contamination are yet to be determined (Kuzman et al., 2014). Mismatched lens case and solution brands resulted in more positive cultures than matched lens care items (Eslami et al., 2020; Wu et al., 2015). The differences in microbial contamination levels may be related to the tested brand (Willcox et al., 2010). However, other studies did not confirm this association (Kratz et al., 2011; Wu et al., 2015). Microbial contamination of CLC was also significantly different in compliant than in non-compliant CL wearers (Kuzman et al., 2014). Compliance with lens care instructions showed improved effectiveness in preventing microbial contamination in numerous studies (Wilson et al., 1990; Larkin et al., 1990; Fleiszig and Efron, 1992; Gray et al., 1995; Szczotka-Flynn et al., 2010). However, some other studies confirmed that microbial infection occurred despite good compliance (Wilson et al., 1990; Stapleton and Dart, 1995). Consumer behaviors may also enhance contamination risks, such as excessive daily lens wear and swimming in the pool with lenses (Kuzman et al., 2014). Male gender was a predictor of CLC microbial contamination due to lower compliance compared with females (Kuzman et al., 2014; Morgan et al., 2011). However, other studies reported contradictory results (Eslami et al., 2020). Hygiene plays a role in preventing contamination, and daily cleaning of CLC with CL solutions (Kuzman et al., 2014). The use of tap water to clean CLC increases the risk of pathogenic GNB contamination (Wiley et al., 2012; Tilia et al., 2014). Recommendations to use multipurpose solutions, hand washing with soap and water, rubbing CLC with clean hands, rinsing CLC but not in tap water, and matching disinfectant solution with CLC brand to reduce microbial contamination of CLC and air drying CLC effectively prevent or decrease microbial bioburden but not biofilm formation (Wilson et al., 1990; Stapleton and Dart, 1995; Willcox et al., 2010; Wu et al., 2011; Tilia et al., 2014; Wu et al., 2015; Waghmare and Jeria, 2022).

The intended lens wear, whether for therapeutic or cosmetic purposes, was also a predictor of microbial contamination. CLC intended for cosmetic use showed higher microbial contamination levels (Yung et al., 2007b).

CL can harbor microorganisms that may be pathogenic, with acceptable tolerability in immunocompetent individuals (Szczotka-Flynn et al., 2010). Patients at risk of developing ocular inflammation and microbial infection include those with advanced age, using immunosuppressive therapy, undergoing surgery, suffering from systemic diseases, and usual CL wearers (Szczotka-Flynn et al., 2010). Data showed that CLC was predominantly contaminated with bacteria, followed by fungi and protozoa (Gray et al., 1995). GNB are the most frequently isolated microorganisms (Gray et al., 1995; Willcox et al., 2010; Wu et al., 2015), including *P*. *aeruginosa* (Devonshire et al., 1993; Gray et al., 1995; Yung et al., 2007a; Yung et al., 2007b), *S*. *marsescens* and other *Serratia* spp. (Larkin et al., 1990; Devonshire et al., 1993; Yung et al., 2007a; Yung et al., 2007b; Wu et al., 2010; Kuzman et al., 2014), *Acinetobacter* spp. (Larkin et al., 1990; Stapleton and Dart, 1995; Boost and Cho, 2005; Yung et al., 2007a; Yung et al., 2007b; Gray et al., 1995; Kuzman et al., 2014), *Enterobacter* spp. (Boost and Cho, 2005; Larkin et al., 1990; Devonshire et al., 1993; Kuzman et al., 2014; Eslami et al., 2020; Wu et al., 2010; Wu et al., 2015; Clark et al., 1994). *P*. *aeruginosa*, in particular, is linked to corneal infections such as microbial keratitis (MK; Wu et al., 2015). Additional GNB, including *Achromobacter* spp., *Stenotrophomonas* spp. *and Delftia* spp., were also isolated from CLC wearers with MK (Mayo et al., 1986; Wiley et al., 2012). *Acinetobacter* spp. are prevalent in bathroom environments, while the source of coliform species such as *E*. *coli* (Yung et al., 2007a; Yung et al., 2007b; Devonshire et al., 1993; Eslami et al., 2020; Kanpolat et al., 1992), *Enterobacter* spp. (Larkin et al., 1990; Devonshire et al., 1993; Clark et al., 1994; Boost and Cho, 2005; Wu et al., 2010; Wu et al., 2015; Kuzman et al., 2014; Eslami et al., 2020), *Klebsiella* spp. (Shintani, 2015; Santos, 2016; Malavi et al., 2018; Cinelli et al., 2019; United States Pharmacopeia, 2020; Gilchrist, 2022), and *Serratia* spp. (Varvaresou et al., 2009; Wu et al., 2010; Kuzman et al., 2014; Wu et al., 2015; Shintani, 2015; Santos, 2016; Akers, 2016; Malavi et al., 2018) can be a lack of hand hygiene due to contamination with fecal material or contact with bathroom aerosols and surfaces (Boost and Cho, 2005). Other recovered GNBs, such as *S*. *maltophilia* (Wu et al., 2015), *Achromobacter xylosoxidans* (Wu et al., 2015), and *Delftia acidovorans* (Wu et al., 2015), led to corneal infiltrative events in CL wearers (Willcox et al., 2010; Wiley et al., 2012; Wu et al., 2015). GPB included CoNS (Kuzman et al., 2014; Wu et al., 2015), as *S*. *aureus* (Boost and Cho, 2005; Yung et al., 2007a; Yung et al., 2007b; Kuzman et al., 2014; Wu et al., 2015), *S*. *epidermidis* (Devonshire et al., 1993; Willcox et al., 2010; Clark et al., 1994; Kanpolat et al., 1992; Eslami et al., 2020), *Bacillus* spp. (Kuzman et al., 2014; Wu et al., 2015), *Corynebacterium* spp. (Kratz et al., 2011), *Diphteroids* (Devonshire et al., 1993; Clark et al., 1994; Eslami et al., 2020), and *fungi*, such as *Chrysosporium* spp., *Penicillium* spp., *and Candida* spp. (Wilson et al., 1990; Gray et al., 1995; Clark et al., 1994; Kanpolat et al., 1992; Kuzman et al., 2014). Regarding protozoa, in 8% of the studies, *Acanthamoeba* spp. was detected in swabbed CLC (Larkin et al., 1990; Devonshire et al., 1993; Gray et al., 1995; Pens et al., 2008; Clark et al., 1994; Kanpolat et al., 1992).

Despite the use of disinfectants, the lens case bioburden remains high (Wu et al., 2015). Contaminated cases showed multiple bacteria and mixed microbial contaminants, including bacteria, fungi, and protozoa (Donzis et al., 1988; Larkin et al., 1990; Gray et al., 1995; Kuzman et al., 2014; Wu et al., 2015; Szczotka-Flynn et al., 2010; Clark et al., 1994; Wiley et al., 2012). Kuzman et al. (2014) reported 4% mixed contamination compared with 55% detected by Gray et al. (1995) and postulated that modern lens care conferred improved effectiveness (Gilchrist, 2022) in preventing microbial contamination, or the discrepancy in results could just be related to the study protocol and sampling methodology (Kuzman et al., 2014). Microbial contamination of CLC contributes to biofilm formation (Dantam et al., 2012) resistant to antimicrobials and multipurpose solutions, leading to ocular infection and even vision loss (Dart, 1997; McLaughlin-Borlace et al., 1998; Szczotka-Flynn et al., 2010). Biofilms are formed despite good hygiene and compliance, contributing to the permanent transfer of pathogenic microorganisms from the CLC to the lens (Szczotka-Flynn et al., 2010; Vijay et al., 2015). Bacterial biofilms were isolated from the CLC of patients with MK (Szczotka-Flynn et al., 2010). Detected microorganisms include *CoNS* (Mayo et al., 1987), *P*. *aeruginosa* (Mayo et al., 1987), and *S*. *marcescens* (Mayo et al., 1987). These organisms constitute a food source for other microorganisms, including *Acanthamoeba* spp., found in CLC or lens care solutions that harbor bacteria and fungi (Mayo et al., 1986; Donzis et al., 1988; Larkin et al., 1990; Devonshire et al., 1993; Pens et al., 2008; Clark et al., 1994; Table 5).

**Table 5:** Documented microbial contamination of home-use CLC.

Rate of MC				Detected microorganisms				Ref.
All types of CLC	SCL case	HCL case	Method of disinfection	GPB	GNB	Fungi	Protozoa	
46%	43%	52%	Heat, Chemical, Peroxide	CoNS*, *Bacillus* spp.*, *Viridans group Streptococcus*, Diphteroids	*S*. *marcescens*, *S*. *liquefaciens*, *P*. *maltophilia*, *P*. *putida*, *E*. *taylorae*, *P*. *stutzeri*, *A*. *calcoaceticus*, *A*. *lwoffi*, *Enterobacter Cloacae*, *K*. *pneumoniae*, *N*. *subflava*, *N*. *sicca*, *Alcaligenes* sp., *M*. *lacunata*	*Fusarium*, *Candida* spp., *Penicillium* spp., *Cladosporium* spp., *Aspergillus* spp., *Phoma* spp.		Donzis et al. (1988)
42%			Heat, Chemical, Peroxide		*S*. marcescens*, *S*. *liquefaciens*, *Acinetobacter* spp., *Klebsiella* spp., *Enterobacter* spp., *and Aeromonas* spp.		*Free-living amoebae*, *Acanthamoeba*, *Vahlkamphia*, *and H*. *vermiformi*.	Larkin et al. (1990)
19%	19%		Chemical, peroxide		*P*. aeruginosa*			Wilson et al. (1990)
53%	66% preserved solutions vs. 100% preservative-free solutions in individuals not advised on proper lens care; 5–10% in individuals advised on proper lens care	64% in individuals not advised on proper lens care vs. 19% in individuals advised on proper lens care	Chemical, Peroxide, Saline, Miscellaneous	*S*. epidermidis*, *Micrococcus* spp.*	*P*. aeruginosa*, *S*. marcescens*., *Klebsiella* spp., *Enterobacter* spp.	*Aspergillus*, *Cladosporium*, *Exophila*, *and Fusarium*		Wilson et al. (1990)
72%			Heat, Chemical, Peroxide	*S*. *aureus*, *Erysipelothrix* spp., *S*. *microaerophilic*, *S*. *sanguis*, *Nocardia* spp.	*A*. *hydrophilia*, *E*. *cloacae*, *C*. *acidovorans*, *C*. *testosteroni*, *F*. *indologenes*, *K*. *oxytoca*, *K*. *ozaenae*, *K*. *planticola*, *K*. *pneumoniae*, *M*. *atlantae*, *M*. *lacunata*, *M*. spp., *O*. *anthropi*, *Proteus* spp., *Providencia* spp., *P*. *alcaligenes*, *P*. *cepacia*, *P*. *paucimobilis*, *P*. *putida*, *S*. *liquefaciens*, *S*. *multivorum*, *E*. *corrodens*.	*C*. *albicans*		Fleiszig and Efron (1992)
57%			Chemical, Hydrogen peroxide, Heat	*S*. *epidermidis*	*Pseudomonas* spp., *E*. *coli*			Kanpolat et al. (1992)
53%	78%	45%	Chemical, Peroxide	*Diphtheroids*, *S*. *epidermidis*, *Bacillus* spp., *Micrococcus* spp.	*S*. marcescens*, *S*. *liquifaciens*, *S*. *plymuthica*, *S*. *odorifera*, *E*. *coli*, *P*. fluorescens*, *P*. *maltophilia*, *Ps*. *acidovorans*, *P*.*aeruginosa*, *P*.*testosteroni*, *P*. *pickeui*, *P*. *luteola*, *P*. *paucimobilis*, *K*. *pneumoniae*, *K*. *oxytoca*, *E*. *cloacae*, *E*. *aerogenes*, *A*. *denitrificans*, *E*. *agglomerans*, *Achromobacter*, *Y*. *internnedia*, *Acinetobacter* spp.*, *F*. *indologenes*, *F*. *multivorum*, *F*. *meningosepticum*, *Flavobacterium* spp., *Ag*. *radiobacter*, *V*. *metschnikovii*, *M*. *phenylpyruvia*, *Pasteurella* spp., *Y*. *enterocolitica*, *C*. *freundii*	Yeast species	*Acanthamoeba* spp., *Hartmanella* spp.	Devonshire et al. (1993)
81%			x	Diphtheroids*, *Bacillus* spp., *Micrococcus* spp.	*Pseudomonas* spp.*, *Xanthomonas* spp., *Serratia* spp.*, *Klebsiella* spp., *Citrobacter* spp., *Alcaligenes* spp., *Acinetobacter* spp.	*Cladosporium* spp., *Candida* spp., *F*. *solani*, *A*.*s versicolor*, *Exophiala* spp., *and Phoma* spp.	*Acanthamoeba* spp., *Naegleria* spp., *Vahlkampfia* spp., *Hartmannella* spp.	Gray et al. (1995)
24%			Chemical		*X*. maltophilia*, *P*. *cepacia*, *S*. liquefacien*s, *and* *S*. plymuthica*			Midelfart et al. (1996)
85%			Chemical, Peroxide	*CoNS*, *S*. *aureus*,	*P*. *aeruginosa*, *Pseudomonas* spp., *Enterobacter* spp.		*Acanthamoeba* sp.	McLaughlin-Borlace et al. (1998)
39%				*S*. *aureus*	*P*. *aeruginosa*, *Pseudomonas* spp.*, A*cinetobacter* spp.*., *Enterobacter* sp., *Serratia* spp., Coliforms*			Boost and Cho (2005)
34%				*S*. *aureus*, *CoNS*	*X*. *maltophilia*, *Ps*. *Aeruginosa*, *Pseudomonas* spp., *Serratia* spp., *Neisseria* spp., *Moraxella* spp., *Flavobacterium* spp., *E*. *coli*, *Acinetobacter* spp., *A*. *xyloxidans*			Yung et al. (2007a); Yung et al. (2007b)
71%						*Acanthamoeba* spp.		Pens et al. (2008)
58%			Chemical, Hydrogen peroxide	CoNS*, *Bacillus* spp.*., *Micrococcus* spp., *Corynebacterium* spp., *P*. acnes*, *S*. *viridans*, *Streptococcus* spp.	*A*. *xylosoxidans*, *A*. *hydrophilia*, *C*. *meningosepticum*, *D*. *acidovorans*, *E*. *aerogenes*, *E*. *cloacae*, *K*. *oxytoca*, *P*. *aeruginosa*, *S*. *marcescens*, *S*. *putrefaciens*, *S*. *maltophilia*	Filamentary fungi*, yeasts		Wu et al. (2010)
76–92%			Chemical, Peroxide	*S*. *aureus*, *S*. epidermidis*, *S*. *hyicus*, *S*. *lugdunensis*, *S*. saprophyticus*, *S*. *viridans*, *Propionibacterium* spp.*, *Micrococcus* spp.*, *Bacillus* spp.*, *Corynebacterium* spp., *Nocardia* spp., *S*. *pneumoniae*	*S*. maltophilia*, *D*. acidovorans*, *S*. marcescens*, *S*. *liquefaciens*, *Achromobacter* group A, *E*. *cloacae*, *K*. *oxytoca*, *P*. *aeruginosa*, *P*. *putida*, *Moraxella* spp., *K*. *pneumoniae*	*Fungi*		Willcox et al. (2010)
		61%		*Staphylococcus* spp., *Lactobacillus*, *Finegoldia*, *Peptoniphilus*, *Peptostreptococcus*, *Anaerococcus*, *Corynebacterium*, *Gemella*, *Abiotrophia*, *Facklamia*, *Granulicatella*	*Achromobacter* spp.*, *Stenotrophomonas* spp.*, *E*. cloacae*, *S*. marcescens*, *E*. *coli*, *E*. *americana*, *Shigella* spp.*, *D*. acidovorans*, *Ps*. *aeruginosa*, *Dialister* spp., *Megasphaera* spp., *A*. *hydrophila*			Wiley et al. (2012)
62%					*Achromobacter* spp., *Stenotrophomonas* spp., *Delftia* spp., *Enterobacter* spp., *Serratia* spp., *Escherichia* spp., *Ewingella* spp., *Shigella* spp., *P*. *aeruginosa*			Kratz et al. (2011)
42%			Chemical, Peroxide	*S*. *aureus*, CoNS*, *Diphtheroids*, *Bacillus* spp., *Corynebacterium* spp.	*Pseudomonas* spp.*, *Enterobacter* spp., *Serratia* spp.*, *P*. *mirabilis*, *K*. *pneumoniae*, *Acinetobacter* spp.	*Chrysosporium* sp., *Penicillium* spp., *C*. *parapsilosis*		Kuzman et al. (2014)
62%				*S*. aureus*, CoNS*, *Micrococcus* sp., *Bacillus* sp.	*Pseudomonas* spp.*, *Klebsiella* spp., *E*. *coli*			Thakur and Gaikwad (2014)
66%				CoNS*, *S*. *aureus*, *Bacillus* spp.*, *Micrococcus* spp.*., *Propionibacterium* spp., *Corynebacterium* spp., Viridans streptococci, *Nocardia* spp., Unidentified Gram-positive rod	*S*. maltophilia*, *A*. xylosoxidans*, *D*. acidovorans*, *S*. marcescens*, *B*. *cepacia*, *C*. *indologenes*, *C*. *meningosepticum*, *E*. *cloacae*, *Moraxella* spp., *P*. *aeruginosa*, *Acinetobacter* spp., *K*. *pneumoniae*, *S*. *paucimobilis*, *P*. *fluorescens*, *P*. *putida*, *Rhizobium radiobacter*, *S*. *liquefaciens*, *S*. *paucimobilis*, *S*. *multivorum*	Molds and yeasts		Wu et al. (2015)
71% silver-impregnated and 82% regular cases			Chemical	*Micrococcus* spp.*, *S*. *aureus*, *S*. epidermidis*, *S*. *haemolyticus*, *S*. *hyicus*, *S*. *lugdunensis*, *S*. saprophyticus*, *S*. *schleiferi*, *Stomatococcus* spp., *S*. *pneumoniae*, *S*. *viridans*, *Planococcus* spp., *Bacillus* spp., *Corynebacterium*, *Propionibacterium* spp.	*A*. *radiobacter*, *Aeromonas* spp., *B*. *cepacia*, *D*. *acidovorans*, *E*. *cloacae*, *E*. *sakazakii*, *Klebsiella oxytoca*, *P*. *fluorescens*, *P*. *putida*, *P*. aeruginosa*, *Raoultella terrigena*, *Serratia liquefaciens*, *S*. marcescens*, *S*. *maltophilia*	Fungi, yeasts		Dantam et al. (2016)
33%			Chemical	*S*. *aureus*, *S*. epidermidis*, Diphtheroid bacilli*, *L*. *monocytogenes*	*P*. aeruginosa*, *E*. aerogenes*, *S*. *maltophilia*, *E*. *coli*, Alcaligenes*			Eslami et al. (2020)

*Most abundant microorganisms.

Clinical significance:

Studies examining contact lens case contamination span from 1986 to 2020, with findings remaining remarkably consistent throughout this period, indicating that contamination is a persistent, unresolved issue despite advances in lens care technology (Donzis et al., 1988; Wilson et al., 1990; Larkin et al., 1990; Devonshire et al., 1993; Gray et al., 1995; Midelfart et al., 1996; Pens et al., 2008; Simmons et al., 1991; Willcox et al., 2010; Kratz et al., 2011; Kuzman et al., 2014; Eslami et al., 2020; Mayo et al., 1986). Although the vast majority of studies included asymptomatic participants, microbial contamination of CLC represents a significant risk factor for ocular inflammation and infection, particularly in susceptible individuals including those with advanced age, immunosuppressive therapy, recent ocular surgery, systemic diseases, and habitual contact lens wearers (Mayo et al., 1987; Gray et al., 1995; Eslami et al., 2020). The clinical relevance is demonstrated by studies showing that identical pathogenic microorganisms, particularly *P*. *aeruginosa*, *S*. *marcescens*, and *Acanthamoeba* spp., are isolated from both contaminated CLC and corneal ulcers in the same patients (Mayo et al., 1987; Gray et al., 1995). Persistent CLC contamination with these organisms is directly associated with MK (Mayo et al., 1987; Donzis et al., 1988; Wu et al., 2015) and sterile corneal infiltrates (Stapleton and Dart, 1995; Donzis et al., 1988; Wiley et al., 2012). Donzis et al. (1988) reported multiple cases of microbial keratitis and diffuse, punctate corneal epithelial opacities specifically attributable to Bacillus spp. contamination of CLC. Of particular concern, biofilms in CLC, especially in areas less exposed to disinfecting solutions such as the upper inner rim (Wu et al., 2010), harbor pathogens resistant to routine disinfection that can contaminate lenses or users’ fingers during handling. These microbial communities persist despite compliance with cleaning protocols and serve as reservoirs for repeated pathogen introduction to the ocular surface (Szczotka-Flynn et al., 2010; Wiley et al., 2012).

##### 3.4.4.2 Toothbrushes

Oral diseases are a growing public health concern, predominantly in low- and middle-income countries (LMICs; Peres et al., 2019). Oral health is the mainstay of systemic health and overall well-being (Peres et al., 2019; Hung et al., 2019; Pradeep et al., 2022). Numerous studies have shown that daily tooth brushing improves oral hygiene (Pradeep et al., 2022; Lee and Lee, 2019; Manohar et al., 2022). Directly after and upon repeated use, the toothbrush becomes infected with various microorganisms (Glass and Lare, 1986; Bunetel et al., 2000; Bonten et al., 1996; Frazelle and Munro, 2012). The microbial contamination sources are the oral microbiome, tap water, the storage surroundings, storage devices, contaminated hands, and aerosols (Pradeep et al., 2022; Lee and Lee, 2019; Manohar et al., 2022; Frazelle and Munro, 2012; Basman et al., 2015). Toilet flushing is a particularly important source of bioaerosols, generating thousands of aerosols per flush that can contain microorganisms from excreta and vomit; these aerosols can remain suspended in the air and settle onto toothbrushes stored in nearby bathrooms (Johnson et al., 2013). Other factors that can influence microbial contamination of toothbrushes include the shape, type of toothpaste used, frequency of brushing, and duration of use (Lee and Lee, 2019). Data have also shown that toothbrushes can be readily contaminated before use (Lee and Lee, 2019; Basman et al., 2015). The bacteria count in a toothbrush varies on average between 10^3^ and 10^5^ CFU per toothbrush (Taji and Rogers, 1998; Basman et al., 2015; Lee and Lee, 2019). Microbial contaminants of toothbrushes were the oral cavity and environmental bacterial flora, pathogenic bacteria, and fungi (Lee and Lee, 2019; Glass and Lare, 1986; Malmberg et al., 1994; Mehta et al., 2007). Multiple studies documented microbial contamination of adult toothbrushes with potential pathogenic bacteria that can cause oral diseases, including *Pseudomonas* spp. (Glass and Lare, 1986; Mehta et al., 2007), MDR *Enterococcus* spp., *Streptococcus* spp., and *Micrococci* spp. (Pradeep et al., 2022; Lee and Lee, 2019). Additional reported bacteria that can cause caries, periodontal, and systemic diseases, included *Neisseria* spp., *Haemophilus* spp., *Bacillus* spp., *E*. *coli*, *S*. *aureus*, *Helicobacter* spp., and *Corynebacterium* spp. (Lee and Lee, 2019; Joy et al., 2022). Other studies documented toothbrushes contamination with *Candida* spp., *Lactobacillus* spp., and *Actinomyces* spp. (Lee and Lee, 2019; Nascimento et al., 2010). Malmberg et al. (1994) examined children’s toothbrushes and found *Staphylococci*, *Streptococcus* spp., *Pseudomonas* spp., *Haemophilus* spp., and fungi (Malmberg et al., 1994).

The American Dental Association (ADA) recommends replacing toothbrushes every 3 months and even more frequently in cases of systemic diseases, ongoing organ transplantation, and chemotherapy (Lee and Lee, 2019; Basman et al., 2015). Studies also mentioned the importance of daily brushing and the appropriate storage of toothbrushes (Pradeep et al., 2022). However, there is no clear guidance on effective toothbrush decontamination or disinfection (Pradeep et al., 2022). Using a toothbrush head (protective cap) can lower microbial contamination (Manohar et al., 2022).

Clinicals significance:

Toothbrushes can serve as a reservoir and a vector for disease transmission in immunocompromised individuals (Pradeep et al., 2022; Lee and Lee, 2019; Frazelle and Munro, 2012). Contaminated toothbrushes can contribute to oral and even systemic diseases, such as cardiovascular, respiratory, gastrointestinal, and renal diseases (Pradeep et al., 2022; Basman et al., 2015; Manohar et al., 2022). Despite these health risks, optimal storage and maintenance conditions for toothbrushes remain poorly defined and unstandardized. Similarly, insufficient guidance exists for the proper care and disinfection of interdental brushes and tongue scrapers, which are equally susceptible to microbial contamination. Dental prostheses, including dentures and partial dentures, also pose contamination risks but lack clear, evidence-based hygiene protocols for home use, particularly regarding cleaning frequency, appropriate disinfection methods, and storage conditions.

## 4 Regulatory frameworks governing product quality and safety

The products examined in Section 3 fall under distinct regulatory classifications with varying definitions across jurisdictions. Understanding these regulatory frameworks is essential for interpreting contamination patterns, establishing appropriate microbiological specifications, and contextualizing industry prevention strategies. Table 6 summarizes key definitions and classification systems for medical devices, medicines, and cosmetics under European and US regulatory frameworks (Sutton, 2018; Fink and Akra, 2023; Van Norman, 2016; Toklu et al., 2019; European Commission, 2007; Food and Drug Administration, 2024, 2025).

**Table 6:** Products definitions and classification.

Product category	EU definition & authority	US definition & authority	Key classification differences
Medicines/Drugs	Substances that treat, prevent, or diagnose disease or restore/correct/modify physiological functions via pharmacological, immunological, or metabolic action (EMA)	Articles for diagnosis, cure, mitigation, treatment, or prevention of disease; affects body structure/function. Includes articles in official pharmacopeias (FD&C Act §201(g))	US includes articles recognized in official Pharmacopeias and formularies; US provides more elaborate legal clauses. The FD&C Act separates the definition into different clauses that cover the intended effects on disease states, body structure/function, and components, providing a more elaborate legal framework. EMA definition specifies mechanism (pharmacological/immunological/metabolic)The EMA’s definition is more generalized, based on the “substance” definition and focused on the pharmacological aspect
Medical Devices	Instruments, apparatus, appliances, software, implants, reagents, materials for medical purposes (MDR 2017/745, Article 2). Includes devices for IVF, conception control, sterilization of other devices. Classes: I (lowest risk), IIa, IIb, III (highest risk)	Instruments, apparatus, appliances, software, implants, materials for diagnosis, treatment, prevention of disease (FD&C Act §201(h)). Classes: I (lowest risk), II, III (highest risk)	EU explicitly includes software, IVF devices, sterilization devices. EU Class II subdivided (IIa, IIb); US has no subdivision
Cosmetics	Substances/mixtures for external body parts, teeth, oral mucous membranes for cleaning, perfuming, appearance alteration, protection, odor correction (Directive 76/768/EEC). One category per product based on function.	Articles for rubbing, pouring, sprinkling, spraying, applying to body for cleansing, beautifying, promoting attractiveness, altering appearance (FD&C Act). Can be both cosmetic AND drug simultaneously.	The definition of Cosmetic products differs across different countries. EU categorizes each cosmetic product into one category based on its intended function and site of application. The FD&C Act defines cosmetics by their intended use.

## 5 Regulatory framework and microbiological standards

Regulatory frameworks govern product approval, manufacturing quality control, and post-market surveillance through distinct mechanisms in the European Union and United States. Microbiological specifications established by these frameworks define acceptable contamination levels for products during manufacturing and distribution. Table 7 outlines the regulatory oversight systems applicable to each product category, providing context for understanding industry prevention strategies and the transition from controlled manufacturing to consumer-use environments (Food and Drug Administration, 2024; Pandey et al., 2019; Regulation (EU) 2017/745, 2017; Fink and Akra, 2023; Directive, 1990; Council, 1993; Culyer, 2014; Brody, 2016; Union, 2009; Ferreira et al., 2022; Halla et al., 2018; Gagliardi and Dorato, 2007; European Commission, 2024; Bernauer et al., 2021; Milstein et al., 2006; Benson and Reczek, 2021; US Code, 2011; FDA, 2022; Huang et al., 2017).

**Table 7:** Regulatory oversights and approval systems.

Product category	EU regulatory framework	US regulatory framework
Medicines	Authority: EMA (established by Regulation 2309/93). EMA coordinates the evaluation of scientific data related to the approval, manufacturing, and inspection of medicines	Authority: FDA (approval and safety oversight per FD&C Act).
	Process: Centralized authorization; pharmacovigilance system tracks adverse effects throughout product lifespan.	Scope: Drugs, food, devices, cosmetics
	Special regulations: Orphan drugs (EC 141/2000), pediatric medicines (EC 1901/2006), advanced therapies (EC 1394/2007).	
	Updates: April 2023 proposal to replace Directive 2001/83/EC and Regulation 726/2004	
Medical Devices	Regulation: MDR (EU 2017/745), implemented May 2021. Replaced MDD (93/42/EEC) and AIMDD (90/385/EEC).	Authority: FDA (sole authority for devices, drugs, biologics).
	Oversight: 38 notified bodies (as of March 2023) overseen by member state competent authorities.	Approval: FDA approval for a medical device does not impose a specific time limit on the duration of the device marketing unless in instances of initiated recalls.
	Approval: CE mark required; validity ~5 years; renewal requires conformity reassessment.	
Cosmetics	Regulation: EC 1223/2009.	Authority: FDA (post-market control under FD&C Act and FPLA).
	Safety Committee: SCCS (Scientific Committee on Consumer Safety); latest guidance May 2023 (12th revision).	GMP: Non-binding guidance (updated 2013 per ISO 22716); compliance not mandatory.
	GMP: ISO 22716 mandatory for all EU products	Industry guidelines: PCPC (Personal Care Products Council)

GMP, Good Manufacturing Practice; ICCR; EMA, European Medicines Agency; International Cooperation on Cosmetic Regulation; MDR, Medical Device Regulation; MDD, Medical Device Directive; AIMDD, Active Implantable Medical Device Directive; PCPC, Personal Care Products Council; SCCS, Scientific Committee on Consumer Safety; FD&C Act, Federal Food, Drug, and Cosmetic Act; FPLA, Fair Packaging and Labeling Act.

Among the product categories examined in this review, cosmetics have the most explicitly defined microbiological quality limits standardized across products within each category. Pharmaceutical products and medical devices are subject to product-specific bioburden or sterility requirements that vary based on risk classification, route of administration, and preservation strategy. Table 8 presents the standardized microbiological specifications for cosmetic products that establish maximum acceptable contamination levels at the point of manufacture (Bernauer et al., 2021; US Code, 2011; FDA, 2022; Huang et al., 2017).

**Table 8:** Microbiological quality limits for cosmetics.

Jurisdiction	Product category	Aerobic mesophilic microorganisms (CFU/g or CFU/mL)	Pathogen limits (*P*. *aeruginosa*, *S*. *aureus*, *C*. *albicans*)	Testing standard
EU	Category 1: Children <3 years, eye area, mucous membranes	≤10^2^ CFU/g	Absent in 1 g or 1 mL	EN ISO 17516:2014
EU	Category 2: All other products	≤10^3^ CFU/g	Absent in 0.1 g or 0.1 mL	EN ISO 17516:2014
US (PCPC)	Eye zone and baby products	<5 × 10^2^ CFU/g	Not specified	Industry guidelines
US (PCPC)	All other products	<10^3^ CFU/g	Not specified	Industry guidelines
US (FDA)	All cosmetics	Not mandatory to be sterile	Should be free from pathogens; limited non-pathogenic organisms	No mandatory standard

EU, European Union; US, United States; FDA, Food and Drug Administration; CFU, colony-forming unit.

## 6 Manufacturing prevention strategies and their limitations in home settings

Given the regulatory frameworks described above, industry has developed comprehensive strategies to prevent microbial contamination during manufacturing and distribution. However, as demonstrated in Section 3, these prevention measures, while effective within controlled manufacturing environments, cannot fully protect products throughout their entire lifecycle once they enter diverse and uncontrolled home settings. Understanding these manufacturing strategies and their inherent limitations is essential to addressing the home-use contamination challenge.

### 6.1 Primary prevention of microbial contamination during the manufacturing process

Microbial contamination of sterile, non-sterile medicines, medical devices, cosmetics, and personal use products may occur during the manufacturing process (Primary contamination; Halla et al., 2018; Bashir and Lambert, 2020), along the supply chain, and during consumer use (Secondary contamination; Halla et al., 2018; Bashir and Lambert, 2020). During the manufacturing process, prevention of microbial contamination begins with securing microbiologically safe raw materials by applying defined specifications and acceptable contamination levels, and following hurdle technology that combines various factors to inhibit microbial growth and preserve product integrity.

#### 6.1.1 Adherence to good manufacturing practice

Industry compliance with GMP guidelines is essential to control microbial contamination (Grazal and Earl, 1997; FDA & EU-GMP, 2018; Food and Drugs Administration, 2019). GMPs are production standards that secure the quality and safety of drugs, medical devices, cosmetics, food, and dietary supplements under industry-standard conditions (Food and Drugs Administration, 2019). Various governments, retailers, consumers, and regulators, including EMA and the US FDA, endorse GMPs (Food and Drugs Administration, 2019). These guidelines cover premises, equipment design and maintenance, employee practices, sanitation, raw material sourcing, production control, recordkeeping, and reporting (Food and Drugs Administration, 2019).

For medicines, GMPs for pharmaceutical drug production include CGMP (drug) in the US and EU-GMP in the European Union (EU; Food and Drugs Administration, 2019; Grazal and Earl, 1997; FDA AND EU-GMP GMP Journal, 2018). The US CGMP is regulated by the FDA (Food and Drugs Administration, 2019). These GMPs ensure manufacturers maintain the identity, strength, quality, and purity of drugs throughout production (FDA AND EU-GMP GMP Journal, 2018). US and EU GMP subject areas are fundamentally similar (Grazal and Earl, 1997). In the US, regulations are more prescriptive and have changed little since 1978, and are less aligned with current science and technology (Grazal and Earl, 1997). EU GMPs are more detailed and enforced by the EMA (Grazal and Earl, 1997). In the EU, a qualified person certifies GMP compliance for each drug batch, for commercial or investigational use (Grazal and Earl, 1997). In the US, the FDA enforces GMP (Grazal and Earl, 1997).

For medical devices, the GMPs are different for sterile and non-sterile products (Food and Drugs Administration, 2019; Grazal and Earl, 1997; FDA AND EU-GMP GMP Journal, 2018). Sterile products require higher levels of cleanliness and control for the prevention of microbial contamination, including sanitation, sterilization, terminal sterilization, aseptic processing, and sterilization by filtration or other processes such as radiation, in addition to guidelines for personnel, premises, and finishing sterile products than non-sterile products (Food and Drugs Administration, 2019; Grazal and Earl, 1997; FDA AND EU-GMP GMP Journal, 2018). Non-sterile products necessitate guidelines for microbiological quality, personnel, premises, equipment, raw materials, and finished products (Food and Drugs Administration, 2019; Grazal and Earl, 1997; FDA AND EU-GMP GMP Journal, 2018). The GMP is mandatory for drugs and medical devices, while adherence to the GMP for personal care products, including cosmetics, varies between countries (Food and Drugs Administration, 2019; Grazal and Earl, 1997; FDA AND EU-GMP GMP Journal, 2018).

For cosmetic products, the US FDA has released non-legally binding, though highly encouraged, regulations and procedures, although manufacturers’ compliance is not obligatory (FDA, 2022; Benson and Reczek, 2021). In Europe, compliance with GMP guidelines for cosmetic products, outlined by the ISO 22716 standard, is mandatory for all products sold in the EU according to the Cosmetics Regulation (EC) 1,223/2009 (CEWAY, 2013; New Directions Aromatics, 2007). In 2013, the FDA updated the GMP guidelines that accounted for the ISO 22716 statements. In 2007, the International Cooperation on Cosmetic Regulation (ICCR) set by the US, Canada, EU, and Japan, recommended the use of this standard in cosmetic GMP guidelines (New Directions Aromatics, 2007). The EU cosmetics GMP– ISO 22716 requirements entail providing guidance for the production, storage, and shipment to maintain the safety and quality of the supply chain (CEWAY, 2013). Cosmetics manufacturers must also ensure the safety, quality, and efficacy of all products (Siegert, 2012; Geis, 2021).

#### 6.1.2 Securing the microbiological quality of raw materials

The microbiological quality of raw materials, including water, is vital in cosmetics manufacturing (Siegert, 2012). Raw material specifications established during procurement define acceptable microbiological limits to ensure quality standards are met from the outset (European Pharmacopoeia Commission, 2023). Contamination of raw materials occurs during transportation, storage, and handling (Elmorsy and Hafez, 2016; Jairoun et al., 2020; Alshehrei, 2023). Raw materials of natural origin (i.e., animal or vegetal) are more likely to be contaminated than synthetic products (Jairoun et al., 2020). Synthetic materials that undergo additional stages during manufacturing may be prone to contamination, such as kaolin, sugar, and vitamins (Halla et al., 2018). Detected contaminants of raw materials predominantly include GNB inspected upon receipt and tested for quality control before use (Jairoun et al., 2020). The European Pharmacopeia provides monographs detailing microbiological quality requirements for raw materials, including acceptable limits for total aerobic microbial count, total combined yeasts and molds count, and absence of specific objectionable microorganisms (European Pharmacopoeia Commission, 2023). Appropriate storage conditions and handling procedures can decrease the risk of cross-contamination between various materials (Siegert, 2012). Water and water-containing raw materials are of concern in cosmetics manufacturing because they can be readily contaminated (Siegert, 2012; Jairoun et al., 2020). Water can be a source of microbial contamination during the cosmetics manufacturing process (Halla et al., 2018; Alshehrei, 2023). The origin of water determines its microbiological quality (Halla et al., 2018). The presence of species such as *E*. *coli* indicates contamination with wastewater (Halla et al., 2018). Numerous species have also been detected in natural water, including *Pseudomonas* spp., *Xanthomonas* spp., *Flavobacterium* spp., *Aeromonas* spp., and *Aerobacter* spp. (Neza and Centini, 2016; Gupta et al., 2024). Appropriate water sterilization and treatment, such as microfiltration, UV light, chlorination, and heat, can be used to prevent contamination (Halla et al., 2018; Geis, 2021; Varvaresou et al., 2009).

#### 6.1.3 Hygiene strategies for personnel, premises, and equipment

The Personnel can be a great source of microbial contamination. The possible reasons are poor hygiene and personal cleanliness, inadequate gowning, lack of training, and malpractice. Microbial contamination can occur through contaminated hands, droplets from coughing, or from applied cosmetics, human skin, hair, oral flora, and even intestinal flora. Reported bacteria include GPB, such as *Staphylococcus* spp., *Micrococcus* spp., *and GNB*, *such as Pseudomonas* spp., *Shigella* spp., *and Acinetobacter lwoffii* (Eissa, 2016).

Microbiological monitoring of the manufacturing facility, including equipment and environment, is critical to prevent microbial contamination during the manufacturing process (Geis, 2021). Poor equipment cleaning and disinfection, maintenance of materials containing oil and grease, can be a source of microbial contamination (Eissa, 2016; Halla et al., 2018). Air and surface quality in manufacturing premises must be controlled according to their intended use and product type, with rooms classified from ISO 8 for general production areas to progressively cleaner classifications (ISO 7, ISO 6) for controlled environments, up to ISO 5 for critical operations such as aseptic processing in pharmaceutical production, sterile medical device manufacturing, and high-risk cosmetic products (ISO, F. N. E, 2015; Abuhav, 2018; Sandle, 2022; Gilchrist, 2022; Whyte, 2024). Equipment may be hard to clean, such as the screw threads (Akers, 2016). Those used for premises mopping and brooming can also be hazardous if not appropriately cleaned (Shah, 2004; Akers, 2016; Medpack, 2023).

While these manufacturing controls are essential, they cannot fully protect products throughout their entire lifecycle. The risk of microbial contamination continues beyond the factory, with potential cross-contamination occurring during storage, transfers, distribution, retail, and consumer use (Ryan, 2017; Lykov and Loboda, 2022), necessitating secondary prevention measures that must function in uncontrolled home environments.

### 6.2 Secondary prevention of microbial contamination

Several strategies are applied to prevent microbial contamination of these products before and during use. However, as evidenced in Section 3, these strategies demonstrate varying degrees of effectiveness once products transition from controlled manufacturing and retail environments to diverse home settings.

#### 6.2.1 Water activity and formulation-based microbial risk

Water activity (Aw) is a critical parameter for microbial preservation in non-sterile pharmaceutical products, defined as the ratio of the water vapor pressure of a formulation to that of pure water, ranging from 1.00 (pure water) to 0.00 (completely dry materials; Varvaresou et al., 2009; Gueye, 2024; United States Pharmacopeia, 2017; United States Pharmacopeia, 2020). Water activity measures the amount of free water available in a product that can support microbial growth and is essential for formulation development, setting microbiological specifications, microbial testing strategies, and risk assessment (Gueye, 2024; United States Pharmacopeia, 2017). Microbial contamination risk is directly linked to water availability in formulations, with different product types presenting varying levels of susceptibility based on their Aw values (Varvaresou et al., 2009; Gueye, 2024). Aqueous formulations with high water activity (Aw >0.95), such as oral liquids, nasal sprays, ophthalmic solutions, and shampoos, create favorable conditions for microbial growth and product stability (Varvaresou et al., 2009; Gueye, 2024). These high Aw products can become breeding grounds for GNB, including the notorious *Burkholderia cepacia* complex, if not properly preserved (Gueye, 2024). In general, bacteria have higher water requirements for growth than yeasts, and yeasts require more water than molds (Schultz, 2016). GNB generally exhibits higher sensitivity to low Aw than GPB (Hiom, 2013; Schultz, 2016). Low water activity can also cause desiccation of microorganisms that can actively reduce the microbial load. This disproportionally affects gram-negative bacteria on dry surfaces (Hiom, 2013; Schultz, 2016). For water-based products, lowering water activity and incorporating preservatives are fundamental preservation strategies (Gueye, 2024; Schultz, 2016).

Dry formulations with low water activity (Aw <0.60), such as tablets, capsules, powders, lyophilized products, and chewable gels, have limited microbial risk due to insufficient free water for microbial growth (European Pharmacopoeia Commission, 2023; Gueye, 2024). When stored in moisture-resistant packaging, these products may remain free from microbial contamination throughout their shelf life and typically do not require the addition of chemical preservatives (United States Pharmacopeia, 2017; Gueye, 2024). However, spore-forming bacteria, including *Bacillus* spp., can survive extreme environmental conditions (Schultz, 2016), including very low Aw environments, and may germinate upon reconstitution or exposure to moisture.

Oily and lipophilic formulations pose different preservation challenges. While low water activity limits microbial growth, these formulations are susceptible to oxidative degradation and may support the growth of lipolytic microorganisms. Additionally, many traditional water-soluble preservatives may not distribute effectively in oil-based matrices (Khanum and Thevanayagam, 2017).

Methods to control water activity include drying (Hiom, 2013; Schultz, 2016), use of vapor-resistant packaging, film strip packing, adding high concentrations of salt or sugar (Hiom, 2013; Schultz, 2016; Halla et al., 2018), and maintaining low Aw through desiccants and individual packaging (Varvaresou et al., 2009; Schultz, 2016; Halla et al., 2018). The FDA Draft Guidance “Microbiological Quality Considerations in Non-sterile Drug Manufacturing” emphasizes that cGMP regulations require stability assessment programs focusing on microbiological control, even for components with low water activity (Gueye, 2024). Appropriate packaging plays an important role in maintaining the intended water activity and preventing moisture ingress throughout the product lifecycle (see section 6.2.4).

#### 6.2.2 pH adjustment and control

The pH level required to prevent microbial growth can vary depending on the formulation (e.g., aqueous liquid form), the types of targeted microorganisms and other factors, such as the temperature and preservative used (Halla et al., 2018; Varvaresou et al., 2009; Vázquez-Blanco et al., 2018). Controlling the pH in pharmaceuticals and cosmetics industry is crucial predominantly to ensure active substance, product stability, microbial stability, and enhance preservative function (Kaple, 2020). For effective preservation, the pH must be maintained within extreme conditions (i.e., less than 4 or greater than 10; Varvaresou et al., 2009). In general, a pH between 5 and 8 offers optimum conditions for the growth of most microorganisms (Halla et al., 2018).

However, extreme pH conditions that would effectively prevent microbial growth (outside pH 6–8) are generally incompatible with pharmaceutical products for multiple reasons: they can compromise preservative chemical stability (particularly at pH > 8), affect drug solubility and stability, and impact product palatability. Additionally, the optimal pH for antimicrobial efficacy often conflicts with the optimal pH for other critical product attributes (Elder et al., 2012). Consequently, most pharmaceutical products require pH compromises that balance microbial control, chemical stability, and product performance. This necessitates the incorporation of additional preservation strategies, particularly the use of preservatives, to prevent microbial contamination at suboptimal pH ranges (see section 5.2.3).

#### 6.2.3 Selection of preservatives

A preservative is a natural or synthetic product added to a cosmetic or pharmaceutical formulation to prevent microbial growth (Dao et al., 2018). An ideal preservative has a broad antimicrobial activity (GNB, GPB, yeast, and mold), low toxicity, high effectiveness at small doses, compatibility with other ingredients in the formulation, stability at a variable range of temperature and pH, and is able to remain in the aqueous phase of a multiphase product (Halla et al., 2018; Dao et al., 2018; Roy et al., 2023). Synthetic preservatives are chosen based on these characteristics, either alone or in combination, to broaden the spectrum of activity, address the risk of toxicity, and prevent microbial resistance (Halla et al., 2018). Natural preservatives are selected according to their properties, including preservative attributes (Halla et al., 2018; Dao et al., 2018; Roy et al., 2023). However, they may pose a significant challenge due to their volatility, strong odor, activity loss due to dilutions, and lipophilic aspects (Halla et al., 2018; Roy et al., 2023). Some preservatives can serve a multifunctional purpose in a product’s functionality and preservation (Halla et al., 2018). Regulatory bodies such as the FDA, EMA, and other national authorities maintain lists of authorized preservatives for use in pharmaceutical and cosmetic products, each with specified maximum concentrations (Union, 2009; Food and Drugs Administration, 2019). Manufacturers must demonstrate through preservative efficacy testing that they have selected the lowest effective dose that provides adequate antimicrobial protection, thereby limiting preservative usage and minimizing potential adverse impacts on consumers and the environment (Halla et al., 2018; Dao et al., 2018). The safety of these products remains a primary concern, mandating thorough evaluation of preservative toxicity and potential adverse reactions (Union, 2009; Halla et al., 2018; Dao et al., 2018). Due to increasing consumer concerns about preservative safety, potential allergenicity, and environmental impact, there is a growing trend toward reducing or eliminating preservatives from pharmaceutical and cosmetic formulations (Porges et al., 2004; Roy et al., 2023). This has driven innovation in alternative preservation strategies, particularly through advanced packaging technologies (see section 6.2.4) that minimize microbial contamination risk through design features such as airless systems, single-dose packaging, antimicrobial packaging materials, and barrier technologies that reduce or eliminate the need for chemical preservatives while maintaining product safety and stability throughout the supply chain and consumer use (Feuillolay et al., 2018; Murray et al., 2019; Iskandar et al., 2022; Liu and ODonovan, 2022; Roquefeuil et al., 2024).

#### 6.2.4 Selection of appropriate primary packaging

Packaging serves as a barrier for product preservation, including protection against microbial contamination and the accumulation of contaminants in the distribution system (Feuillolay et al., 2018). The type of packaging of pharmaceutical and cosmetic products depends on the ingredients and the intended use (Feuillolay et al., 2018).

Optimal packaging should provide physical, chemical, and microbiological stability of the product along the supply chain and during consumer use (Feuillolay et al., 2018; Halla et al., 2018; Roy et al., 2023). From a preservation perspective, single-dose or unit-dose packaging represents the ideal choice as it eliminates repeated exposure to environmental contamination and user contact. However, the significantly higher costs and environmental impact associated with unit-dose packaging make multi-use packaging the predominant choice despite the increased risk of microbial contamination through repeated opening and product withdrawal (Reis et al., 2025). The physical and chemical stability of the product is the mainstay for the determination of its shelf-life, predominantly in the pharmaceutical industry (Feuillolay et al., 2018). An ideal packaging must be non-leaching, environmentally friendly, and should protect from moisture and environmental conditions, such as temperature, humidity, and light (Cinelli et al., 2019; Liu and ODonovan, 2022; Roy et al., 2023). Critically, the packaging materials and components themselves must not be contaminated with microorganisms. Current industry standards require microbiological controls and testing of packaging materials before use to ensure they do not introduce contamination into the final product (Feuillolay et al., 2018). An example of such a product is airless packaging (Varvaresou et al., 2009). Jars and bottles are more prone to microbial contamination than closed-system items such as compressed gases, pumping configurations, and the use of narrow-opening containers (Yablonski and Mancuso, 2004; Varvaresou et al., 2009; Halla et al., 2018). The use of re-closable containers, the size of the packaging, the delivery holes, and the container influence microbial contamination (Brannan and Dille, 1990; Song et al., 2003; Zema et al., 2010; Halla et al., 2018). The type of closure also shows variable protection against microbial contamination (Gad et al., 2021). A study showed that the screw-cap exhibited minimal protection, while the flip-cap and pump-top closures provided better preserving effect than the slit-cap (Brannan and Dille, 1990). The tip of the container also offers a surface prone to microbial contamination (Varvaresou et al., 2009; Wiley et al., 2012; Iskandar et al., 2022). The product withdrawal zone (dispensing area, opening, tip) has been identified as a major risk area for microbial contamination, as it experiences repeated contact with the user’s hands, skin, and environmental surfaces (Varvaresou et al., 2009; Dao et al., 2018). To address this critical risk, innovative packaging solutions incorporating antimicrobial activity in high-risk zones have been developed. These include packaging components with integrated antimicrobial agents such as silver (Ag) ions and/or nanoparticles and antimicrobial mineral microspheres specifically applied to dispensing tips, closures, and product contact surfaces materials (Liu and ODonovan, 2022; Reis et al., 2025). These antimicrobial mineral technologies provide continuous protection against microbial contamination at the most vulnerable points of product contact, potentially reducing or eliminating the need for high concentrations of chemical preservatives while decreasing the bioburden on surfaces throughout the entire product lifecycle.

The different manufacturing prevention strategies, from GMP compliance to advanced packaging technologies, the home-use contamination patterns documented in Section 3 reveal the fundamental challenge: the myriad variables introduced when products enter uncontrolled consumer environments should lead to a combination of preventive measures to ensure a significant reduction in microbiological risk. The gap between manufacturing quality assurance and home-use realities necessitates additional approaches, including consumer education, product design innovations specifically accounting for home-use conditions, and, as discussed in the following section, understanding that even pre-market contamination remains a persistent industry challenge.

## 7 Pre-market contamination: manufacturing and distribution challenges

While Section 6 outlined the comprehensive strategies employed to prevent contamination during manufacturing, these measures do not secure total preservation. Pre-market contamination, occurring during manufacturing, storage, or distribution before products reach consumers, remains a persistent industry challenge, as evidenced by product recalls. Understanding the scope and nature of pre-market contamination provides important context for distinguishing it from the secondary, post-market contamination documented in Section 3. This distinction is critical: pre-market contamination represents manufacturing and quality control failures, whereas post-market home-use contamination occurs despite products initially meeting microbiological specifications.

### 7.1 Product recall due to microbial contamination

Product recalls in the cosmetic and pharmaceutical industry are necessary to enforce quality standards and ensure public safety (Natof and Pellegrini, 2021). Recalls can occur due to various issues, including labeling errors, product defects, and detection of microbial contaminants (Wang et al., 2012; Natof and Pellegrini, 2021). Microbial contamination may be visible (e.g., mold growth) but can also lead to other product modifications (e.g., odor, texture). Thus, microbial contamination is a significant concern in these industries, as it may lead not only to user dissatisfaction but to health problems and even death (Heneghan et al., 2011; Kramer et al., 2012; Eissa, 2016), especially in vulnerable populations such as children and the elderly (Jimenez, 2019; Miglani et al., 2022; David and Jimenez, 2022).

In the cosmetic industry, microbial contamination is a common cause of product recall (Food and Drug Administration, 2024). Between 2005 and 2018, 104 reports of microbiologically contaminated cosmetics with approximately 20% intended for children (Michalek et al., 2019). The most common contaminants were GNB, primarily *Pseudomonas* spp. (Babalola and Eze, 2015) and *Enterobacter* spp. (Babalola and Eze, 2015; Michalek et al., 2019), which may be resistant to preservatives found in cosmetic products and can result in infections (Periame et al., 2014; Periame et al., 2015; Neza and Centini, 2016; Michalek et al., 2019). Other contaminants included GPB such as *Bacillus firmus*, *Enterococcus* spp., and *S*. *aureus*, fungi like *C*. *albicans* (Babalola and Eze, 2015), and additional yeasts and molds (Michalek et al., 2019).

In the pharmaceutical industry, GNB, yeasts, and molds are the most common contaminants in medical devices and sterile and non-sterile medications (Jimenez, 2007; Jimenez, 2019). *B*. *cepacia* is a particularly problematic contaminant in both sterile and non-sterile drugs (Jimenez, 2007; Singhal et al., 2015; Marquez et al., 2017; Becker et al., 2018; Jimenez, 2019). *Salmonella* spp. was reported as a contaminant of raw materials and non-sterile drug products (Jimenez, 2019). Other microbial contaminants in non-sterile pharmaceutical products included *Klebsiella* spp., *E*. *coli*, *Pseudomonas* spp., and GPB contaminants, predominantly *Staphylo*coccus spp. (Jimenez, 2019). Fungal contamination is increasingly reported as the cause of pharmaceutical product recalls (Sandle, 2014), with few reports providing information at the genus or species level (Sandle, 2014; Jimenez, 2019; David and Jimenez, 2022). Non-sterile product contamination included *Candida* spp., *Aspergillus* spp., and *Penicillium* spp. (Miglani et al., 2022). A study conducted by Jimenez (2019) analyzed the FDA enforcement reports from 2012 to 2019 and revealed that 87% of microbial contaminants of sterile drug products remain unidentified (Jimenez, 2019). Contaminants of sterile products included Aspergillus spp. and GNB, suggesting a water system problem during manufacturing, while identified GPB, including *Bacillus* spp. *and Staphylococcus* spp., indicated environmental control issues (Jimenez, 2019). The primary cause of microbial contamination remains the lack of sterility assurance, although law enforcement has recently become more stringent (Jimenez, 2019).

The pre-market contamination patterns described above, characterized by manufacturing failures, preservation system inadequacies, and quality control lapse, differ fundamentally from the post-market contamination documented in Section 3. Pre-market contamination typically involves isolated batches or manufacturing errors that trigger recalls, whereas home-use contamination represents a systematic, widespread phenomenon affecting products that initially met all microbiological specifications. This critical distinction indicates that the home-use contamination problem cannot be addressed solely through improved manufacturing practices; it requires managing consumer storage, handling, and use behaviors in uncontrolled home environments.

## 8 Discussion

Despite comprehensive Good Manufacturing Practices, advanced preservation systems, and robust regulatory frameworks, microbial contamination of home-use medicines, medical devices, cosmetics, and personal care products remains a widespread, underrecognized threat to public health. Perhaps the most concerning finding across product categories is biofilm formation documented in nebulizers (Jarvis et al., 2014), contact lens cases (Bell et al., 2020; Jarvis et al., 2014; Hutchinson et al., 1996; Dao et al., 2018; Tabatabaii et al., 2020; Blau et al., 2007; Yegit et al., 2025), feeding bottles (Rachon et al., 2017; Rothstein et al., 2019), pacifiers (Comina et al., 2006), and toothbrushes. Biofilms confer remarkable resistance to disinfection, facilitate persistent colonization, and serve as reservoirs for repeated reintroduction of pathogens into vulnerable anatomical sites (Hutchinson et al., 1996; Dart, 1997; McLaughlin-Borlace et al., 1998; Cohen et al., 2006; Keen et al., 2010; Szczotka-Flynn et al., 2010; Foreman and Wormald, 2011; Foreman et al., 2011; Jervis-Bardy et al., 2011; Tan et al., 2014; Jarvis et al., 2014).

Across product categories, standardized cleaning guidance for home users remains fragmented and product-specific. No universal protocol exists. Breast pumps have the most robust guidance, with the CDC publishing detailed cleaning and sanitizing protocols (Centers for Disease Control and Prevention, 2024). Contact lens care products are covered by ISO 14729, which establishes microbiological requirements for commercial care solutions, though this does not address user cleaning behaviors. For nebulizers, the European Respiratory Society has explicitly noted the absence of ideal standards and called for a universal code of practice (Bell et al., 2020). For cosmetic applicators such as makeup brushes, standards such as ISO 21322 exist but are designed for manufacturer testing, not consumer cleaning guidance. Across all categories, consumers are left to follow variable manufacturer instructions, highlighting a critical gap requiring evidence-based, standardized home cleaning protocols. The growing consumer demand for preservative-free and “natural” products (Liu and ODonovan, 2022; Roy et al., 2023) creates tension with contamination prevention needs. While preservative elimination reduces potential allergic reactions and environmental impact, it increases microbial risk, particularly in high water activity formulations (Varvaresou et al., 2009; Hiom, 2013; Schultz, 2016; United States Pharmacopeia, 2017; United States Pharmacopeia, 2020; Gueye, 2024). This preservation paradox necessitates alternative strategies beyond traditional chemical preservatives.

Innovative packaging technologies offer promising solutions. Airless systems, single-dose packaging, and antimicrobial materials incorporated into product contact surfaces provide protection without relying solely on chemical preservatives (Varvaresou et al., 2009; Iskandar et al., 2022; Roquefeuil et al., 2024). The prevention of home-used products microbial contamination relies on enhancing consumers’ awareness of the risks and educating them and their families about infection prevention measures. However, engaging and empowering the patient or consumer to be the partner at home for infection prevention is challenging (Donskey, 2023). Numerous socio-economic and socio-cultural barriers may influence sustainable adherence to effective preventive measures (Ward, 2011; Tsang and Vayalumkal, 2016; Zakar et al., 2021; Mirfardi, 2023). Multiple studies highlighted these challenges by examining the influence of the social determinants of health interplays on adherence to infection prevention measures (Tziraki-Segal et al., 2019; Shushtari et al., 2021; Lipshutz et al., 2022).

Antimicrobial materials and surfaces represent emerging technologies that contribute to minimizing and preventing microbial contamination (Song et al., 2003; Brannan and Dille, 1990; Zema et al., 2010; Feuillolay et al., 2018; Cinelli et al., 2019; Iskandar et al., 2022; Roquefeuil et al., 2024). A critical distinction exists between antimicrobial surfaces that rely on controlled release of antimicrobial substances and those that provide antimicrobial activity without releasing active agents. For contact lens cases specifically, antimicrobial agents including silver, polyquats, selenium, copper, zinc nanocoating, and quorum-sensing blockers have demonstrated varying efficacy (Ryan, 2017; Dao et al., 2018; Feuillolay et al., 2018; Cinelli et al., 2019; Liu and ODonovan, 2022; Reis et al., 2025). Ideally, non-leaching, sustainable green technologies that are patient and environment-friendly are needed (Bharadwaj and Dutta, 2021). Recent studies showed that mineral microspheres incorporated in eye drop bottle tips provide contact-based antimicrobial activity without releasing agents, substantially decreasing surface contamination (Roquefeuil et al., 2024). Similar non-leaching technologies are being incorporated in cosmetic products, contributing to polymer reduction and recyclable, bio-based materials (Somater, 2023). Multiple antimicrobials with different mechanisms remain under investigation. However, a combination of approaches appears necessary to significantly reduce microbiological risks.

Immunocompromised individuals, neonates, elderly persons, and patients with chronic diseases face disproportionate risks from contaminated home-use products (Shintani, 2015; Jimenez, 2019; Miglani et al., 2022; Tropea, 2022; Roy et al., 2023; Osuoha et al., 2023; da Silva et al., 2025; Tyski et al., 2025). These populations have heightened susceptibility to opportunistic pathogens commonly isolated from contaminated products.

Health equity considerations are paramount. Contamination risks are amplified in socioeconomically disadvantaged and resource-constrained households,where water quality, access to appropriate disinfectants, and educational resources are limited (Cherian and Lawande, 1985; Imong et al., 1995; Redmond et al., 2009; Gibson et al., 2017; Rachon et al., 2017; Marege et al., 2023). The lack of consumer awareness, particularly in susceptible individuals, increases infection risk (Ferrer and Klein, 2015; Gaube et al., 2019). Health literacy gaps further contribute to these risks, as misinterpretation or neglect of manufacturer cleaning instructions can lead to improper dilution, insufficient contact times, or unsafe mixing of disinfectants, reducing efficacy and elevating microbial hazards (Wolf et al., 2005). Health risk perception is crucial in individuals’ engagement with preventive behaviors and adherence to public health measures (Ferrer and Klein, 2015; Gaube et al., 2019). Research indicates that risk perception formation involves deliberative, affective, and experiential components that interactively influence health behaviors (Ferrer and Klein, 2015). Understanding these dynamics is essential for developing targeted public health communications and interventions that effectively promote prevention measures across diverse populations and health contexts (Ferrer and Klein, 2015; Gaube et al., 2019). Significant knowledge gaps persist despite extensive literature. First, methodological heterogeneity across studies limits comparability, contamination definitions, sampling techniques, detection methods, and reporting standards vary widely. Regarding detection methods, most studies relied on conventional microbiological culture, with identification based on colony morphology, Gram staining, and biochemical tests. Only a minority employed molecular techniques such as 16S rRNA sequencing or PCR for species confirmation. Some studies targeted specific pathogens of interest (e.g., *P*. *aeruginosa*, *S*. *aureus*), potentially missing other clinically relevant organisms. Agar choice and incubation conditions also influence which organisms are recovered. These methodological differences directly affect reported contamination rates and organism profiles, as culture-based methods may underestimate fastidious or uncultivable organisms while molecular methods may detect non-viable or environmental DNA. Standardized protocols are urgently needed to enable meaningful cross-study comparisons. Second, most studies examined asymptomatic users, with limited data linking contamination patterns to clinical outcomes. Longitudinal studies establishing causality between product contamination and infection incidence are lacking. Third, the directionality of contamination (patient-to-device vs. device-to-patient) remains unclear for many products (Wexler et al., 1991; Hutchinson et al., 1996; Cobben et al., 1996; Saiman and Siegel, 2004; Brzezinski et al., 2011; Manor et al., 2017; Tabatabaii et al., 2020). Fourth, biofilm assessment remains inconsistent across studies despite its clinical importance. Fifth, contamination thresholds associated with infection risk are poorly defined for most products. Sixth, geographical disparities in contamination patterns require investigation (Psaltis et al., 2012). Finally, emerging concerns including antimicrobial resistance in home-contaminated products, microbiome alterations from contaminated devices (Moossavi et al., 2019), and safety of homemade/DIY cosmetics (Couteau et al., 2022) lack adequate research attention.

The findings and recommendations of this review align with several United Nations Sustainable Development Goals (SDGs; United Nations, 2015). Protecting vulnerable populations, neonates, immunocompromised individuals, and the elderly, from infections caused by contaminated home-use products directly supports SDG 3 (Good Health and Well-being), which calls for ensuring healthy lives and promoting well-being for all at all ages. The disparities in contamination risk highlighted in this review, particularly the amplified hazards in low-resource settings due to limited access to clean water, appropriate disinfectants, and health literacy, reflect the objectives of SDG 10 (Reduced Inequalities). The advocacy for non-leaching, recyclable, bio-based packaging technologies and the reduction of chemical preservatives align with SDG 12 (Responsible Consumption and Production), which promotes sustainable resource use and responsible business practices. Furthermore, the need for innovative antimicrobial surface technologies, biofilm-resistant materials, and green packaging solutions underscores the objectives of SDG 9 (Industry, Innovation and Infrastructure), which emphasizes building resilient infrastructure and fostering innovation. Advancing these goals in tandem is essential for developing sustainable, equitable, and effective strategies to mitigate the hazard of microbial contamination in home-use products.

### 8.1 Strengths and limitations

This comprehensive narrative review synthesized evidence across diverse product categories, regulatory frameworks, and contamination patterns, encompassing 346 peer-reviewed sources. The cross-product approach revealed common contamination mechanisms and risk factors not apparent in category-specific reviews. However, limitations exist. As a narrative rather than systematic review, selection bias is possible. Study heterogeneity precluded meta-analysis. English-language restriction and publication bias toward positive findings may limit generalizability. Data from LMICs are underrepresented. Variable contamination thresholds and detection methods across studies complicate direct comparisons.

## 9 Conclusion

Microbial contamination of home-use medicines, medical devices, cosmetics, and personal care products represents a significant yet underrecognized threat to public health, particularly among vulnerable populations. A paradigm shift from reactive contamination management to proactive prevention is urgently needed, requiring coordinated action across multiple fronts. Regulatory bodies must establish post-market surveillance systems monitoring real-world contamination patterns and extend safety frameworks beyond manufacturing to consumer use. Industry must prioritize innovative product designs incorporating antimicrobial surfaces, airless packaging, biofilm-resistant materials, and single-dose formats, particularly using green technologies. Healthcare providers require evidence-based, standardized hygiene protocols that are practical and accessible to diverse user populations. Public health interventions must account for risk perception dynamics, socioeconomic barriers, and health equity considerations. Despite limited evidence regarding optimal educational strategies, bundled interventions can be implemented (Agreli et al., 2019; Food and Drug Administration, 2024). Only through integrated efforts across regulatory, manufacturing, healthcare, and public health sectors can the consumers be adequately protected from microbial hazards in their homes, thereby advancing both public health and sustainable development goals globally.

## Glossary

GMP: Good Manufacturing Practices
CRS: Chronic rhinosinusitis
GNB: Gram-Negative Bacteria
CoNS: Coagulase-negative staphylococci
ESS: Endoscopic Sinus Surgery
CF: Cystic fibrosis
GPB: Gram-Positive Bacteria
COPD: Chronic Obstructive Pulmonary Disease
CDC: Centers for Disease Control and Prevention
CHILD: Canadian Healthy Infant Longitudinal Development
HIV: Human Immunodeficiency Virus
DYI: do-it-yourself
CLC: contact lens cases
CL: Contact lens
MK: Microbial keratitis
LMICs: Low- and middle-income countries
PCPC: Personal Care Products Council
EU: European Union
US: United States
FDA: Food and Drug Administration
CFU: Colony Forming Unit
MDD: Medical Device Directive
AIMDD: Active Implantable Medical Device Directive
SCCS: Scientific Committee on Consumer Safety
MDR: Medical Device Regulation
EMA: European Medicines Agency
FD&C Act: Federal Food, Drug, and Cosmetic Act
FPLA: Fair Packaging and Labeling Act
